# Potential demand for voluntary community-based health insurance improvement in rural Lao People’s Democratic Republic: A randomized conjoint experiment

**DOI:** 10.1371/journal.pone.0210355

**Published:** 2019-01-08

**Authors:** Thiptaiya Sydavong, Daisaku Goto, Keisuke Kawata, Shinji Kaneko, Masaru Ichihashi

**Affiliations:** 1 Graduate School for International Development and Cooperation, Hiroshima University, Higashi-hiroshima, Hiroshima, Japan; 2 Department of Planning and Investment, Savannakhet Provincial Government, Savannakhet, Lao People’s Democratic Republic; 3 Institute of Social Science, University of Tokyo, Tokyo, Japan; Brown University, UNITED STATES

## Abstract

**Introduction:**

In Lao People’s Democratic Republic (PDR), community-based health insurance (CBHI) is the only voluntary insurance scheme; it typically targets self-employed people, most of whom reside in rural areas and are dependent on agricultural activities for subsistence. However, until very recently, the enrollment rate has fallen short and failed to reach a large percentage of the target group. To promote the CBHI scheme and increase demand, some supporting components should be considered for inclusion together with the health infrastructure component.

**Objectives:**

This paper provides empirical evidence that the benefit package components of hypothetical CBHI schemes have causal effects on enrollment probabilities. Furthermore, we examine the distribution of willingness to pay (WTP) in response to policy changes based on a sample of 5,800 observations.

**Methods:**

A randomized conjoint experiment is conducted in rural villages in Savannakhet Province, Lao PDR, to elicit stated preference data. Each respondent ranks three options—two hypothetical alternatives and the CBHI status quo scheme. The levels of seven attributes—*insurance coverage for medical consultations*, *hospitalizations*, *traffic accidents*, *pharmaceuticals* and *transportation*; *premiums*; and *prepaid discounts*—are randomly and simultaneously assigned to the two alternatives.

**Results:**

The findings suggest that the average WTP is at least as large as 10.9% of the per capita income of those who live in rural areas, which is higher than the WTP for health insurance averaged across low- and middle-income countries (LMICs) in the literature. The component of round-trip transportation insurance coverage has a significant effect on WTP distribution, particularly increasing the share of the highest bin.

**Conclusion:**

Therefore, the low CBHI scheme enrollment rate in Lao PDR does not necessarily imply low demand among the targeted population, as the finding from the WTP analysis illustrates potential demand for the CBHI scheme. Specifically, if transportation is addressed, enrollment is likely to significantly increase.

## Introduction

Community-based health insurance (CBHI) schemes are a risk-pooling system that have received increasing attention as a powerful tool for health system improvement, particularly in terms of financial protection and health equity, in LMICs [1]. Scheme enrollment is on a voluntary basis, and the pooling of health risk and prepayment typically occur at the community level. Under the risk-pooling system, individuals’ financial burden is spread across all scheme members, making health care more affordable for the poor. Therefore, beneficiaries are protected against the catastrophic costs of illness while ensuring their right to equal access to health services based on their needs.

Although this scheme aims to reduce reliance on direct out-of-pocket (OOP) expenditures and to facilitate the targeted population’s utilization of health services, in practice, the implementation of this scheme is slow and laborious, especially in low-income countries. According to an extensive body of empirical work, the four common problems in CBHI scheme implementation are low enrollment rates [2, 3], adverse selection [4–7], poor quality of healthcare [8], and high drop-out rates [9–11]. The low enrollment rates of targeted populations are considered not only the primary challenge to the financial sustainability of the scheme but also an indicator of low acceptance of the scheme [12]. In particular, the literature often reports disappointing enrollment percentages, with the percentage of the eligible population covered varying between 1% and 10% [3, 13–15] for most cases and, rarely, between 21% and 46% [16, 17].

The low popularity of CBHI schemes among targeted populations is a major concern for governments in low-income countries. To design relevant measures to encourage enrollment, policy practitioners need to return to those factors that might influence the targeted population’s demand for health insurance. The evidence from observational studies of the determinants of CBHI membership can be summarized as follows: premium unaffordability, limited healthcare facilities, long distance to healthcare facilities, insufficient information, poor quality of health services (including out-of-stock drugs), and ordinary benefit packages might be bottlenecks leading to low enrollment rates [11, 18–23]. These studies suggest that among the four problems, healthcare quality and benefit packages should be prioritized for improvements to promote CBHI enrollment. However, the following questions remain: how should benefit packages be designed to meet the preferences of potential enrollees? What is potential enrollees’ average WTP for health insurance?

In general, WTP can be estimated by two approaches: revealed preference, which is based on actual behavior in response to actual policy, and stated preference (SP), which is based on hypothetical behavior in response to hypothetical policy [24]. SP is more applicable to the study of potential policy improvement. There are two methods for collecting SP: the contingent valuation method (CVM) and discrete choice experiments (DCEs). Specifically, in the CVM, respondents are directly asked about their WTP for a hypothetical policy. This method has been widely applied in the field of health economics [25–28]. A recent systematic review of the application of the CVM to measure WTP for health insurance in LMICs concluded that the average WTP for health insurance of rural households is less than 2% of GDP per capita [29]. However, the CVM can only observe the value of a policy as a complete bundle.

By contrast, DCEs are an attribute-driven experimental method in which a hypothetical policy is described by a set of attributes, including pecuniary attributes. In DCEs, usually, potential products or services are described by their characteristics, referred to as attributes, and each attribute is assigned a range of defined dimensions called attribute levels. Each attribute is assigned a fixed position throughout all choice sets, referred to as attribute positions. Therefore, DCEs can be used to measure the value of each policy attribute and produce findings that are more informative for policy interventions [30]. Due to this obvious advantage, DCEs have received dramatically more attention in the literature, particularly the health economics literature that addresses health-related policy concerns [31]. In LMICs, a number of studies have utilized DCEs to either quantify individual preferences or to measure individuals’ WTP for a health insurance intervention, such as micro health insurance in the health care system in Liberia [32] and Malawi [33], social health insurance in Ethiopia [34], CBHI in Cambodia [16], and social health insurance in Bulgaria [35]. The selection of attributes (and attribute levels) for DCEs in these studies was rather diverse across settings. Frequently considered attributes include the health insurance premium, the availability of medicines, transportation-related attributes (for instance, insurance coverage for transportation to healthcare facilities or distance to health care facilities), enrollment units, copayments, wait times, and reputation of the healthcare staff. From the health system perspective, these attributes are all prerequisites for effective improvements; however, in practice, these policy interventions are very costly and have many limitations, in particular, budget constraints. Therefore, to prioritize and design relevant improvement measures considering the preferences of potential enrollees, it is important to identify the causal effect of each attribute in isolation on their choice probabilities. Although DCEs can elicit the effect of changes in individual policy attributes on the WTP distribution, they cannot identify the causal effects of individual policy attributes on choice outcomes unless a complete set of interventions is conducted.

To address the above limitations of conventional DCEs, Hainmueller et al. (2014) developed a new conjoint analysis based on the potential outcome framework for causal inference [36]. The conjoint analysis requires a fully randomized design in which attribute levels and attribute positions are randomly assigned and alternatives are randomly paired. This fully randomized design ensures that the observable and unobservable confounding variables of the respondents are approximately equally distributed across the treatment assignments (choice tasks). Therefore, the conjoint analysis with the fully randomized design enables researchers to estimate the nonparametric causal effect of each attribute component based on the observed outcome. An increasing number of studies have applied this approach to estimate the average marginal component effect (AMCE) in a variety of contexts, for instance, the causal effects of immigrant attributes on Americans’ attitudes towards immigrants [37] or the causal effects of different climate policies on public support [38–40].

An outstanding empirical example of the application of this approach is the study of Hninn et al. (2017) [41], which was the first study to introduce the theoretical framework on welfare implications using the conjoint experiment of Hainmueller et al. (2014) [36]. Hninn et al. (2017) incorporated the nonparametric point-identification welfare analysis of Bhattacharya (2015) with the observed data from the conjoint experiment approach of Hainmueller et al. (2014) [36, 42]. Unlike the conventional welfare analyses using DCEs, which assume a specification for the choice probabilities so that the results of the welfare analysis are potentially based on the parametric assumption [43], the approach of Hninn et al. (2017) is based on a rational choice model with no need to assume the functional form of the preference distribution [41]. With only weak assumptions regarding the preference distribution, the authors showed that the marginal component effect on the WTP distribution can be nonparametrically measured. The nonparametric analysis is more advanced, in that it addresses the likely misspecification bias in choice making. The first empirical result of this WTP distribution analysis was achieved in a study that evaluated a water quality improvement intervention in Inlay Lake, Myanmar.

To provide more informative potential solutions to the phenomenon of low enrollment in the CBHI scheme with a focus on improving the benefit package, the present study employs the WTP distribution analysis of Hninn et al. (2017) [41]. To date, no studies have applied this approach to the health system context with a focus on improving health financing policy. To ensure effective benefit package design, it is important to evaluate how potential enrollees’ CBHI scheme preferences can be enhanced via a set of attributes and how much they are willing to pay for CBHI scheme improvement.

An advantage of our approach is the simple linear regressions. For instance, conventional DCE analyses are based on logit regression, which assumes a type 1 extreme value distribution. In contrast, our approach is based on simple difference-in-means estimators, which do not require any functional assumptions and are easily implemented by ordinary least squares regressions. Therefore, our estimation results are more valid than those of a conventional DCE analysis.

## Community-based health insurance scheme in Lao PDR

In Lao PDR, health risk is expected to increasingly threaten the poor in particularly remote areas [44], where the majority of the population remains dependent on agricultural activities for subsistence and the infrastructure is inadequate. Therefore, the government is concerned with strengthening the health system—health financing schemes in particular—to ensure health equity for all groups in the population.

To improve the health system, the government launched four health financing schemes targeting specific groups in the population, including State Authority Social Security (SASS) for government workers, the Social Security Organization for salaried employees of private and state-owned enterprises, Health Equity Funds (HEFs) for the extremely poor, and CBHI for workers in the informal sector [45]. Among the four schemes, only the CBHI scheme is based on voluntary membership and is implemented in a decentralized manner.

As of 2014, only 27.2% of the population was covered by any scheme within the health financing system. Moreover, the decomposed coverage by scheme is rather heterogeneous. While the coverage of the SASS and HEF schemes, which target nearly 26.5% of the Lao population, achieved approximately 85% of target, the coverage of the HEF and CBHI schemes showed little progress, with only 6.4% of the targeted group enrolled. In particular, the CBHI scheme, which targets approximately two-thirds of the Lao population, achieved only 3.7% of the target by 2014. In other words, the CBHI scheme has the largest target but the lowest achievement. This study chooses to evaluate the CBHI scheme for three main reasons: 1. the scheme is voluntary, 2. the targeted population is mainly the poor in rural areas with limited infrastructure and geographic constraints, and 3. the scheme has made extremely slow progress towards its given target.

In 2002, the Ministry of Health introduced the CBHI scheme as a pilot project in two districts, with technical assistance from the WHO and financial support from the United Nations Human Security Fund. As of September 2015, the scheme was available in 50 of the 148 districts in 17 of the 18 provinces, which is equivalent to 2,271 of the 8,507 villages. The total number of beneficiaries is reported to be 33,795 households (179,534 people). Currently, the benefit package of the CBHI scheme covers outpatient and inpatient services, including primary health care, specialist services, diagnostic tests, and prescribed pharmaceuticals that are available in hospitals. The household is the unit of enrollment, and the premiums vary depending on urban or rural residence and the number of household members. The premium rates have not been updated since 2005 [46]. The window period of service access is three months upon enrollment. With the gatekeeping system, CBHI members have to first seek services at contracting facilities, such as dispensaries and district hospitals, and can only be sent to provincial or regional hospitals with a referral [47]. Since 2012, 50% of the scheme’s revenue has come from collecting premiums, and the other 50% has come from government subsidies (Decree on national health insurance, 2012).

As mentioned in the Introduction, low enrollment and quality of healthcare are two of the four main concerns that hinder the successful implementation of the CBHI scheme in LMICs, and Lao PDR is no exception. To move towards the goals of this program, it is necessary to improve the enrollment rate and service quality. It is suggested that more CBHI members would induce more financial protection and more cost recovery, which could lead to improvements in service quality [48]. This plan is consistent with a move towards universal health coverage (UHC), which has been defined as the required outcome of health system performance, whereby all people who need health services receive them without excessive financial hardship [49, 50]. The CBHI enrollment ratio indicates the population coverage, which is one of the three dimensions (population coverage, service, and direct cost) of the UHC cube. To achieve the goal of UHC by 2025 through implementation of a risk-protection scheme, which is a commitment of the Lao government, Lao PDR would need to extend coverage to more people, offer more services, and/or pay a greater share of the cost. To this end, this study focuses on addressing the low enrollment problem: we look for potential ways in which the number of enrollees can be increased given the quality of healthcare. Thus, in this context, improvement is limited to benefit package developments such that the scheme is affordable and meets the preferences of the poor. In regard to the existing CBHI benefit package and the condition of its targeted population, we hypothesize that, to some extent, the factor of transportation affects CBHI scheme promotion in rural Lao PDR. We further hypothesize that the enrollment rate can be increased by improving the benefit package and considering potential new incentives. To test the hypotheses, we conduct an experiment that introduces several hypothetical CBHI schemes based on the attributes presented below.

## Methods

### Ethics statement

This study was approved by the Ethical Committee of the Graduate School for International Development and Cooperation of Hiroshima University, Japan, and the District Administration Offices of Champhone and Xaibouly, Savannakhet Province, Lao PDR. Oral informed consent was obtained from all the respondents after the survey objectives and procedures were explained. The respondents were assured that their participation was voluntary and that they could withdraw from the survey at any time. To maintain high confidentiality of the data and protect the respondents’ anonymity, we assigned a unique ID code to each respondent’s data and separated personal identification information from the response data. All sets of data were password protected and saved in different places.

### Design

In designing the experiment, we needed to consider some relevant potential attributes, specifically those attributes that are highly correlated with common CBHI scheme-related problems either in LMICs or in the Lao context. For this study, we started with the reviewed literature and the views of local CBHI staff as relevant and reasonable information sources for attribute selection.

In this study, the hypothetical extended CBHI benefit package is described according to seven attributes: *monthly premiums*; *one-year prepaid discounts*; and *insurance coverage for medical consultations*, *hospitalizations*, *traffic accidents*, *pharmaceuticals*, and *transportation*. Two attributes, insurance coverage for pharmaceuticals [32, 34] and transportation, are selected based on information obtained from the literature review. In particular, according to a systematic review of factors affecting the voluntary uptake of CBHI schemes in LMICs, travel and transport factors could be facilitators of or barriers to access to healthcare at the facilities contracted by CBHI schemes [19]. More specifically, two studies found that long distances from home to healthcare facilities become a barrier to scheme enrollment [51, 52]. In addition, another two studies suggested that low enrollment was caused by high transport expenditure [53, 54], and two conventional DCE studies in Cambodia and Malawi showed that greater insurance coverage of transportation cost strongly influenced CBHI enrollment [16, 33]. However, the existing literature does not identify the causal effect of insurance coverage of transportation cost on the enrollment probabilities of the poor in remote areas. Therefore, this study hypothesizes that transportation cost can be a significant determinant of self-employed people’s reluctance to participate in the CBHI scheme in Lao PDR, where the transportation infrastructure remains a challenging issue in many parts of the country, especially in remote areas.

The attributes of insurance coverage for traffic accidents and prepaid discounts are selected based on interviews with local CBHI staff. The lack of coverage for the medical treatment of injuries due to traffic accidents has long been a complaint of many active CBHI enrollees and former enrollees. Meanwhile, premium collectors at the community level also face difficulties collecting monthly premiums; they state that offering annual collection at a discounted rate might be more relevant to rural enrollees who earn their income on a seasonal basis.

Although the CBHI status quo scheme covers hospitalization and medical consultation fees, we intentionally include these attributes in the experiment to observe how people trade-off between them and other hypothetical attributes.

Table 1 presents the attributes and levels applied in our experiment. The number of attributes and levels generate a total of 575 possible scenarios—excluding the status quo scenario. These scenarios can be combined into 328,902 possible pairs. For simplicity and to maintain the condition of uncorrelated attributes, we randomly select 575 pairs and form 115 choice sets. Each respondent is presented with a set of five choice tasks; thus, the causal effects are estimated using 115 choice sets in total. Note that the reference category of premiums shown in Table 1 refers to the current premiums for those residing in rural areas.

**Table 1:** Attributes and levels.

No.	Attribute	No.	Levels	Description
1	Premiums	1	10,000 LAK (1 member)	Premium per household per month in Lao currency (LAK). It is 2,000 LAK less than current premium, which varies across household size.
			18,000 LAK (2-4 members)	
			23,000 LAK (5-7 members)	
			26,000 LAK (8+ members)	
		2	*12,000 LAK (1 member)*	Premium per household per month in Lao currency (LAK). It is the current premium which varies across household size.
			*20,000 LAK (2-4 members)*	
			*25,000 LAK (5-7 members)*	
			*28,000 LAK (8+ members)*	
		3	14,000 LAK (1 member)	Premium per household per month in Lao currency (LAK). It is 2,000 LAK higher than current premium, which varies across household size.
			22,000 LAK (2-4 members)	
			27,000 LAK (5-7 members)	
			30,000 LAK (8+ members)	
		4	16,000 LAK (1 member)	Premium per household per month in Lao currency (LAK). It is 4,000 LAK higher than current premium, which varies across household size.
			24,000 LAK (2-4 members)	
			29,000 LAK (5-7 members)	
			32,000 LAK (8+ members)	
2	Medical consultations	5	No	Insurance does not cover medical consultations or diagnostic test fees.
		6	*Yes*	Insurance covers medical consultations and diagnostic test fees.
3	Hospitalizations	7	No	Insurance does not cover hospitalization fee for medical treatment or surgery.
		8	*Yes*	Insurance covers hospitalization fees for medical treatment and surgery.
4	Traffic accidents	9	*No*	Insurance does not cover medical treatment fees for traffic accidents.
		10	Yes	Insurance covers medical treatment fees for traffic accidents.
5	Pharmaceuticals	11	*Partly*	Insurance covers fee for pharmaceuticals identified on the essential medicines list of the Ministry of Health.
		12	Fully	Insurance covers all fees for pharmaceuticals associated with treatment.
6	Transportation	13	*No*	Insurance does not cover patients’ cost of travel to referral hospitals out of the district.
		14	One way	Insurance covers patients’ one-way travel cost to referral hospitals out of the district.
		15	Round trip	Insurance covers patients’ round-trip travel cost to referral hospitals out of the district.
7	Prepaid discounts	16	*No*	No discount for a one-year premium prepayment.
		17	5%	5% discount for a one-year premium prepayment.
		18	10%	10% discount for a one-year premium prepayment.

Note: The italic levels are those representing the status quo CBHI scheme.

To minimize bias as a result of the communication process, the survey is carried out in two parts: a household survey and an experiment. First, five investigators conduct questionnaire-based interviews to collect households’ demographic information and self-reported reasons for not enrolling. Second, respondents who complete the first session proceed to the experimental session with a different investigator. Before progressing to the second session, three investigators meet face-to-face with the respondents to explain the scenario and the rules of the experiment and to show them the pictures used in the experiment.

Each respondent encounters five randomly formed choice tasks. In each choice task, the participants compare three policy alternatives, two hypothetical policies and the CBHI status quo scheme and rank the policies based on which scheme will maximize their benefit (1, 2, and 3 indicates the most, average and least preferred policies, respectively). To avoid bias resulting from the order in which attributes are presented, the position order is randomized. For simplicity, a respondent receives the identical attribute order across the five choice tasks.

To ensure that respondents can understand our experimental setup, we conduct a pretest of 20 random samples (equivalent to 200 observations) with individuals of different CBHI statuses—members (households that are enrolled as of September 2016), ex-members (households that dropped out in September 2016), and non-members (households that never enrolled in the CBHI scheme). Note that these 20 households are excluded from the main survey results. The pretest confirms that the selected attributes and the variation in premium levels are relevant and that the design of the experiment is understandable. It further confirms that the number of choice tasks is tiring for respondents, but the tasks are manageable because we utilize pictures. Thus, the bias associated with the effects of illiteracy can be overcome.

### Sampling

This study collects data on the SP of rural households in Savannakhet Province, which has the largest land area and population size. According to a Center National Health Insurance Bureau report, in 2015, Savannakhet Province had the largest number of CBHI members and the greatest fluctuation in membership among all provinces. For sample selection, in this study, the districts and villages are chosen purposely, but the representative households are randomly sampled as follows.

There are 15 districts in Savannakhet Province. Since 2014, eight of the districts have reported increasing numbers of CBHI-enrolled households, while the remaining districts have faced a decreasing number of CBHI members. Note that the provincial capital district needs to be removed from our selection because its infrastructure differs from that of the other districts. To ensure that the results account for the views of heterogeneous respondents, we intentionally select two districts respectively with increasing and decreasing numbers of CBHI members. Accordingly, we choose Champhone and Xaibouly Districts, which have the largest coverage of CBHI respectively among increasing and decreasing districts. However, CBHI coverage in Champhone and Xaibouly Districts accounted for only 0.21% and 0.1% of the province population in 2015, respectively.

As our focus is households in remote areas, to ensure that the experiment can plausibly be conducted in these areas, we purposively designate only type II villages with a homogeneous infrastructure surveillance of 1 1 0 1 1 1 0 (according to Lao Statistics Bureau, villages are classified into three types: village type I indicates an urban village with road access, electricity, water supply, regular market, and administrative office; village type II is a rural village with road access; and village type III is a rural village without road access. The 1 1 0 1 1 1 0 condition indicates road access, electricity; no health care facility; clean water, village drug kits, primary school; and no regular market). Ultimately, we identify three villages in Champhone District and six villages in Xaibouly District. However, one village in Xaibouly District is removed due to accessibility constraints.

All informal-sector households, which are the targets of the CBHI scheme, are eligible for this study. However, in practice, we purposely omit monks because interviews with them are implausible. Members of the eligible population are stratified into three groups: CBHI active members, non-members, and ex-members. Member respondents are randomly drawn from a list of currently active CBHI members in each village, whereas ex-members are randomly selected from a list of those who dropped out before September 2016. Non-members are randomly selected from a list of households in each village excluding those that work in formal sectors and those identified as members or dropouts. Ultimately, there are 580 stratified random samples, representing 46% of the eligible population. Our samples comprise 210 (36%), 72 (13%), and 298 (51%) active members, ex-members, and non-members, respectively.

We exclusively identify the household head or spouse as the representative of the household in the experiment. In the local context, the household head or spouse is the key decision-maker over the allocation of economic resources within the household, and exploring their preferences might result in acceptable and successful health insurance interventions in the future. However, only 88.45% of respondents are household heads or spouses.

The survey was conducted by the research team, Sydavong and Goto. For the household survey, eight local interviewers were employed (three interviewers were staff members of the Provincial Statistics Center who have experience with national surveys, and the other five were recent university graduates with experience conducting household surveys from their own social science studies).

The interviewers were divided into two teams and trained for two days. On the first day, the first team was trained as questionnaire-based interactive interviewers to collect demographic and socioeconomic information about the households. First, the trainer (Sydavong) explained the motivation, objectives and significance of this study in detail to engage the interest of the interviewers in the survey and motivate them. Second, the trainer gave detailed explanations of the survey procedure, informed consent, each question and the method of coding the respondents’ answers. Third, the interviewers were trained in survey techniques, such as how to provide the necessary information, how to ensure the receipt of oral informed consent, how to ask sensitive questions and how to smoothly present questions in a sequence to retain the respondent’s concentration. Any queries and potential confusion were freely shared among the team and the trainer.

On the second day, the second team (who understood DCEs and the CVM as scientific methods) was trained as field experimenters. Similar to the members of the first team, the members of the second team were first informed about the motivation, significance, and objectives of this study. Then, they were introduced to the randomized conjoint experimental techniques. They were informed of the status quo of the CBHI scheme in Lao PDR and the aim of this study: to investigate the stated preferences of respondents towards hypothetical CBHI schemes defined by seven selected attributes. The team was thoroughly informed of the attributes and attribute levels used in this experiment—especially the attribute of the premium, which is rather complicated. They also received an explanation of the scenario for this experiment, the number of options the respondents had for selecting rankings in a choice task, and the number of choice tasks they had to complete. The trainer then demonstrated how to rank the options using the sample. To ensure that the interviewers understood the experiment, the trainer asked the interviewers to rank three options of the five choice tasks based on their preferences and to discuss the reasons. Any queries and potential confusion were freely shared among the team and the trainer.

The main survey was conducted between 13 and 27 September 2016 over the course of two days per village, on average. The participants were recruited and gathered with the assistance of the village chiefs.

### Analysis

#### Estimation of choice probabilities

This paper applies the full randomization design for the conjoint analysis [36]. In this approach, respondents are presented with a set of choice tasks offering several alternatives. Each alternative is randomly formed by a set of selected attributes and individual attribute levels.

For our experiment, we randomly generate two hypothetical CBHI alternatives, each of which is described by the premium and a set of non-pecuniary attributes: insurance coverage for medical consultations, hospitalizations, traffic accidents, pharmaceuticals, and transportation as well as a prepaid discount. Respondents are asked to rank the alternatives based on their preferences.

In this study, we intentionally include the CBHI status quo scheme as one alternative; thus, each choice task has three alternatives: two hypothetical CBHI alternatives and the current CBHI scheme. For this reason, two types of results can be simultaneously observed: the internal choice probability (when an alternative is preferred to another alternative) and the external choice probability (when an alternative is preferred to the status quo).

To view this more formally under Hninn et al.’s (2017) framework [41], let **K** = {1, …, *k*} be a set of choice tasks of CBHI alternatives; *C*_*ijk*_ is the insurance premium; **A**_*ijk*_ = {*A*_*jk*1_, …, *A*_*jkL*_} is a set of non-pecuniary attributes for individual *i* in the *j*^*th*^ alternative of the *k*^*th*^ choice task; and *L* is the number of attributes. Thus, CBHI alternatives are defined by *C*_*ijk*_ and **A**_*ijk*_.

In each of **K** choice tasks, individual *i* chooses between *J* (= 2) hypothetical CBHI alternatives and the CBHI status quo scheme. *Y*_*ijk*_ is the individual choice outcome; if individual *i* prefers alternative *j* in choice task *k* over the status quo (or over the other alternative for internal choice probabilities), *Y*_*ijk*_ = 1 and 0 otherwise.

There are two main assumptions needed for this approach. First, the independence assumption ensures that the round of the choice task and the order of alternatives do not influence the individual choice outcome. Thus, *Y*_*ijk*_, *C*_*ijk*_, and **A**_*ijk*_ can be referred to as *Y*_*ij*_, *C*_*ij*_, and **A**_*ij*_. Second, the randomization assumption allows us to define the AMCE identification of changes in the level of attribute *l* from *a*_0_ to *a*_1_ with the following equation:

$${\hat{\pi }}_{l}\left(a_{1},a_{0}\right)={\bar{Y}}_{ij|C_{ij}=c,A_{ijl}=a_{1}}-{\bar{Y}}_{ij|C_{ij}=c,A_{ijl}=a_{0}}$$ (1)

where *c*, *a*_1_ and *a*_0_ are the given levels of premium (*C*_*ij*_) and attribute *l* (*A*_*ijl*_) for the hypothetical CBHI alternative. ${\bar{Y}}_{ij|C_{ij}=c,A_{ijl}=a_{1}}$ and ${\bar{Y}}_{ij|C_{ij}=c,A_{ijl}=a_{0}}$ are the conditional averages of the observed choice outcome.

According to the aforementioned identification, the AMCE can then be estimated with our conjoint experiment data by using the following linear model:

$$E\left[Y_{ij}\right]=\beta_{0}+\beta_{c}I_{ijc}+\sum_{l=1}^{6}\beta_{l}I_{ijl}$$ (2)

where *E*[*Y*_*ij*_] is the expected binary choice indicator of respondent *i* for CBHI alternative *j*. *I*_*ijc*_ and **I**_*ijl*_ denote the vectors of the dummy for the levels of premiums and the *l*^*th*^ attribute. *β*_*c*_ and *β*_*l*_ are vectors of the estimates of the AMCE. As the experiment allows each respondent to rank three alternatives in several choice tasks, Eq (2) is estimated by the cluster robust standard errors estimation method, accounting for within-respondent correlations between preferences.

#### Component effect on WTP distribution

For the WTP analysis, we mimic the methodology of Hninn et al. (2017) [41]. Four assumptions for the utility function—monotonicity, continuity, boundary, and rationality—are sufficient to identify the marginal WTP distribution as follows:

$$\hat{F}\left(c\right)=1-{\bar{Y}}_{ij|C_{ij}=c}$$ (3)

where $\hat{F}\left(c\right)$ is the identification result of the marginal WTP distribution or the share of those having a WTP value of *c* or lower.

However, the boundary assumption only assumes the lower bound of WTP at zero and does not assume the upper bound [41]. This assumption means that the levels of the non-pecuniary attributes in the alternatives are greater relative to the levels of the CBHI status quo scheme. Therefore, only the lower bounds of the average marginal WTP from the conjoint data can be identified using the following equations:

$$\underline{\hat{\mu }}=\sum_{i=0}^{n}c_{i}\left[\hat{F}\left(c_{i+1}\right)-\hat{F}\left(c_{i}\right)\right]$$ (4)

where *c*_*i*_ is the lower premium in the *i*^*th*^ threshold, and *n* is the number of threshold levels.

Based on the CBHI status quo scheme, in which the premium varies across household size, the hypothetical premium levels in this study also vary accordingly. For simplicity, we define the premium levels as:

$$\begin{array}{ccc} & p_{1}=\{\begin{array}{cc} 10,000\;\;\text{LAK} & \;1\;\text{member}\\ 18,000\;\;\text{LAK} & \;2-4\;\text{members}\\ 23,000\;\;\text{LAK} & \;5-7\;\text{members}\\ 26,000\;\;\text{LAK} & \;8+\;\text{members} \end{array},\; & p_{2}=\{\begin{array}{cc} 12,000\;\text{LAK} & \;1\;\text{member}\\ 20,000\;\text{LAK} & \;2-4\;\text{members}\\ 25,000\;\text{LAK} & \;5-7\;\text{members}\\ 28,000\;\text{LAK} & \;8+\;\text{members} \end{array}\\ & p_{3}=\{\begin{array}{cc} 14,000\;\;\text{LAK} & \;1\;\text{member}\\ 22,000\;\;\text{LAK} & \;2-4\;\text{members}\\ 27,000\;\;\text{LAK} & \;5-7\;\text{members}\\ 30,000\;\;\text{LAK} & \;8+\;\text{members} \end{array},\; & p_{4}=\{\begin{array}{cc} 16,000\;\;\text{LAK} & \;1\;\text{member}\\ 24,000\;\;\text{LAK} & \;2-4\;\text{members}\\ 29,000\;\;\text{LAK} & \;5-7\;\text{members}\\ 32,000\;\;\text{LAK} & \;8+\;\text{members} \end{array} \end{array}$$

From Eq (3), the probability intervals of the marginal WTP distribution are rewritten as:

$$\begin{array}{cc} \hat{F}\left(p_{1}\right) & =1-{\bar{Y}}_{ij|C_{ij}=p_{1}}\\ \hat{F}\left(p_{2}\right)-\hat{F}\left(p_{1}\right) & ={\bar{Y}}_{ij|C_{ij}=p_{1}}-{\bar{Y}}_{ij|C_{ij}=p_{2}}\\ \hat{F}\left(p_{3}\right)-\hat{F}\left(p_{2}\right) & ={\bar{Y}}_{ij|C_{ij}=p_{2}}-{\bar{Y}}_{ij|C_{ij}=p_{3}}\\ \hat{F}\left(p_{4}\right)-\hat{F}\left(p_{3}\right) & ={\bar{Y}}_{ij|C_{ij}=p_{3}}-{\bar{Y}}_{ij|C_{ij}=p_{4}}\\ 1-\hat{F}\left(p_{4}\right) & ={\bar{Y}}_{ij|C_{ij}=p_{4}} \end{array}$$ (5)

Again, the boundary assumption enables us to identify five threshold premium levels, as follows: [0, *p*_1_]; [*p*_1_, *p*_2_]; [*p*_2_, *p*_3_]; [*p*_3_, *p*_4_]; and [*p*_4_, ∞). Thus, the lower bound of the average marginal WTP in Eq (4) is measured as follows:

$$\begin{array}{c} \underline{\hat{\mu }}=(\begin{array}{cccc} 10,000s_{1\;} & 12,000s_{1\;} & 14,000s_{1\;} & 16,000s_{1\;}\\ 18,000s_{2-4} & 20,000s_{2-4} & 22,000s_{2-4} & 24,000s_{2-4}\\ 23,000s_{5-7} & 25,000s_{5-7} & 27,000s_{5-7} & 29,000s_{5-7}\\ 26,000s_{8+} & 28,000s_{8+} & 30,000s_{8+} & 32,000s_{8+} \end{array})(\begin{array}{c} \hat{F}\left(p_{2}\right)-\hat{F}\left(p_{1}\right)\\ \hat{F}\left(p_{3}\right)-\hat{F}\left(p_{2}\right)\\ \hat{F}\left(p_{4}\right)-\hat{F}\left(p_{3}\right)\\ 1-\hat{F}\left(p_{4}\right) \end{array}) \end{array}$$ (6)

where *s*_1_, *s*_2-4_, *s*_5-7_, and *s*_8+_ are the percentages of households that have 1 member, 2-4 members, 5-7 members, and 8 or more members, respectively.

## Results and discussion

### Descriptive statistics

A comparison of the characteristics of our samples with those of samples in rural areas from the Lao Expenditure and Consumption Survey, 2012-2013 (LECS V) shows that our samples tend to be poorer, older, and less educated and to have a larger household size. This result is unsurprising because only informal-sector households in type II villages are eligible for our study. In this study, the respondents are of an average age of 44.66 years, and 58% are female. Of the respondents, 66% completed primary school, 19% secondary school, 13% upper school, and 2% higher education. The average household size is 5.92. The average annual household income is 15.26 mill.LAK (median income: 9.31 mill.LAK). The average annual per capita income is 2.81 mill.LAK, with a right-skewed distribution and a standard deviation of 4.53 mill.LAK. Moreover, 61% of households have an income level below the poverty line (according to the Prime Minister’s Office (2009), a household is not poor if the per capita income is over 180,000 LAK per month, which is the national poverty line in rural areas), and 8% of households report having household members with a chronic disease.

### Model estimation

#### Component effect on choice probabilities

**Fig 1:**
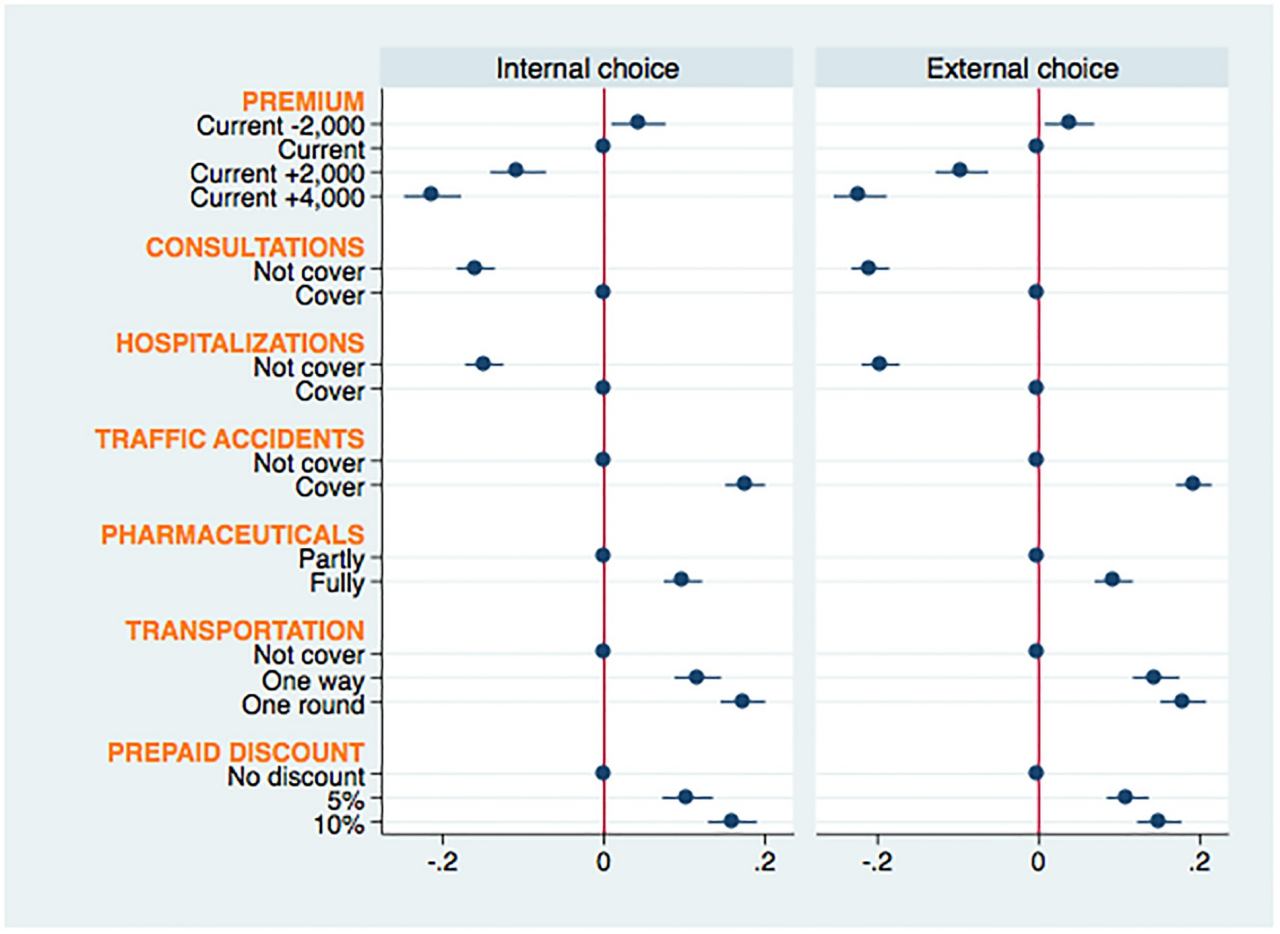
Average marginal component effect on choice probabilities.

Fig 1 shows the results of the AMCE for the internal and external choice probabilities from estimating Eq (2). The dots indicate point estimates of the AMCE for each attribute level indicating the respondents’ choice probability of joining the CBHI scheme compared to the baseline level, and the error bars illustrate 95% confidence intervals. The solid dots along the vertical axis are the reference categories of each attribute.

Overall, the findings show that the signs, magnitudes and significance levels of the estimates are particularly close between the two results, suggesting that the respondents’ preferences are robust. All estimated coefficients are statistically significant at a 99% confidence interval except for that for the current premium, which is significant at a 95% confidence interval. The premium clearly stands out as the major burden affecting the choice probabilities; the roughly monotonic effects of the premium support the monotonicity assumption. The preferences are more sensitive to a higher premium than to a lower premium. In particular, when the presented premium is 4,000 LAK more than the current premium, the probability of joining the CBHI scheme decreases by approximately 21 percentage points compared to the probability given the current premium. This result confirms the findings of previous studies [55] and is consistent with the self-reported reasons why respondents do not enroll in or drop out of the CBHI scheme; 82% of the respondents reported that the inability to pay was their first constraint.

As stated in the experimental design section, we intentionally include the attributes of insurance coverage for consultations and hospitalizations (which are covered in the CBHI status quo scheme) in the experiment to examine their causal effects in comparison with the hypothetical attributes. The resulting internal choice probabilities indicate that the effects of removing either consultation or hospitalization insurance coverage from the CBHI benefit package are roughly less than the effects of including either traffic accidents or round-trip transportation insurance coverage but, on the other hand, are larger than the external choice probabilities. Among the hypothetical attributes (one-year prepaid discounts and insurance coverage for traffic accidents, pharmaceuticals and transportation), coverage for traffic accidents and round-trip transportation had remarkably greater causal effects on the choice probability than the other attributes. This finding can be interpreted as showing that a CBHI scheme that includes insurance coverage for either traffic accidents or round-trip transportation would be more popular among the targeted population. For the external choice probabilities, we can further explore the distribution of respondents’ WTP across the given premium ranges for the CBHI scheme improvements compared to the status quo. The results of Eq (5) are reported in the following section.

#### WTP distribution

**Fig 2:**
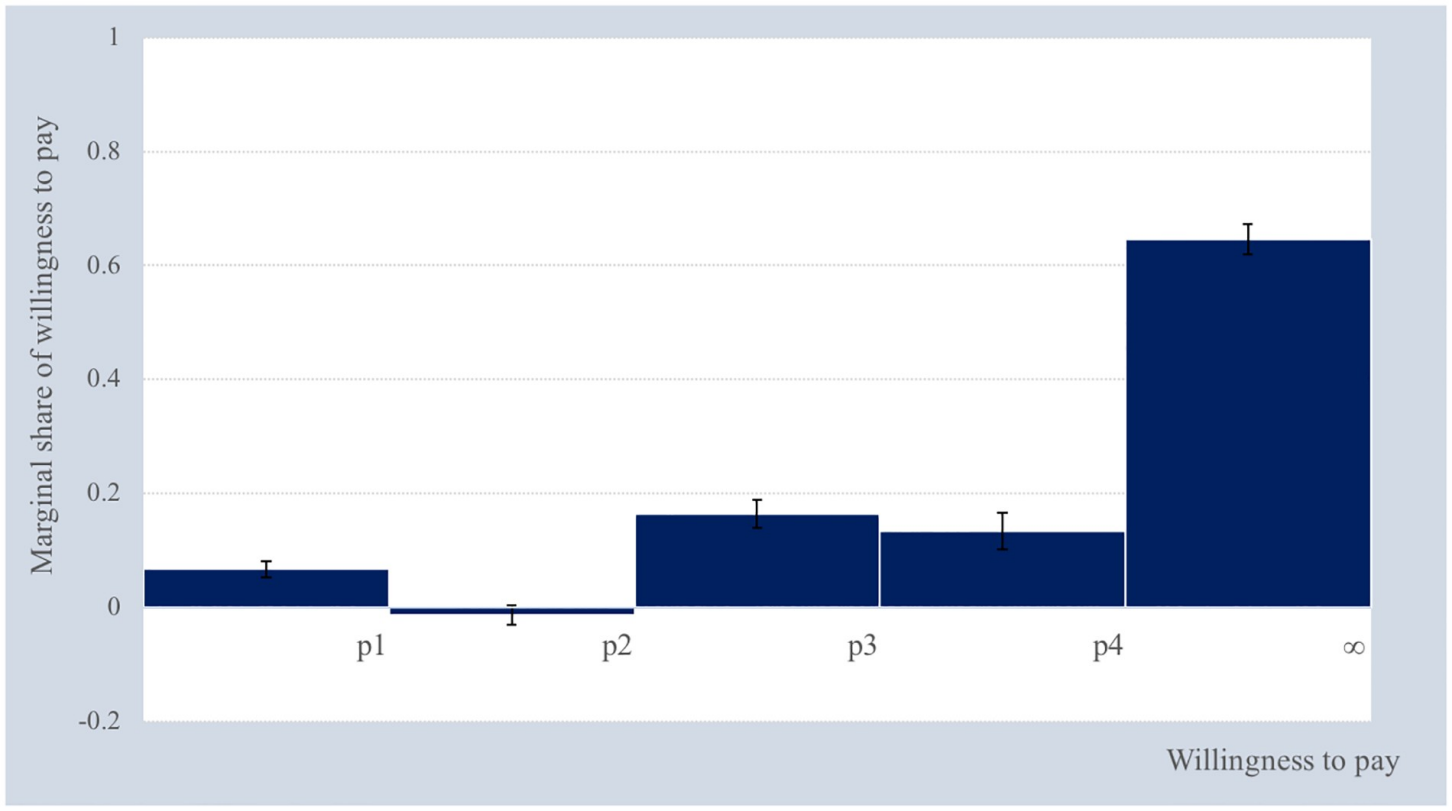
Marginal share of willingness-to-pay.

To estimate the lower bound of the average WTP, we conduct a subsample analysis in which all levels of non-pecuniary attributes meet the boundary assumption. To ensure that the hypothetical alternatives will improve the CBHI status quo scheme, we need to exclude the observations in which the levels of the medical consultation and hospitalization attributes are below the status quo of the CBHI scheme. According to the four levels of premium designed in the conjoint experiment and the boundary assumption, the estimated WTP is distributed into five intervals. Fig 2 shows the marginal share of respondents whose WTP value lies below the upper bound of a certain interval. The error bars display the 95% confidence intervals. The findings show that the estimated marginal WTP values are all significant at the 1% level except that of the second interval, which is negative but nonsignificant. Only 6.74% of the respondents expressed that they are willing to pay only if the premium is at or below *p*_1_. Interestingly, the share of respondents whose WTP value is greater than *p*_4_ is considerably higher than that of the other bins, with a share of 64.61%. Based on Eq (6), the lower bound of the average WTP is measured at 25,579 LAK, which is equivalent to 10.9% of the monthly per capita income of households in the area (or 3.29% of the monthly median household income).

**Fig 3:**
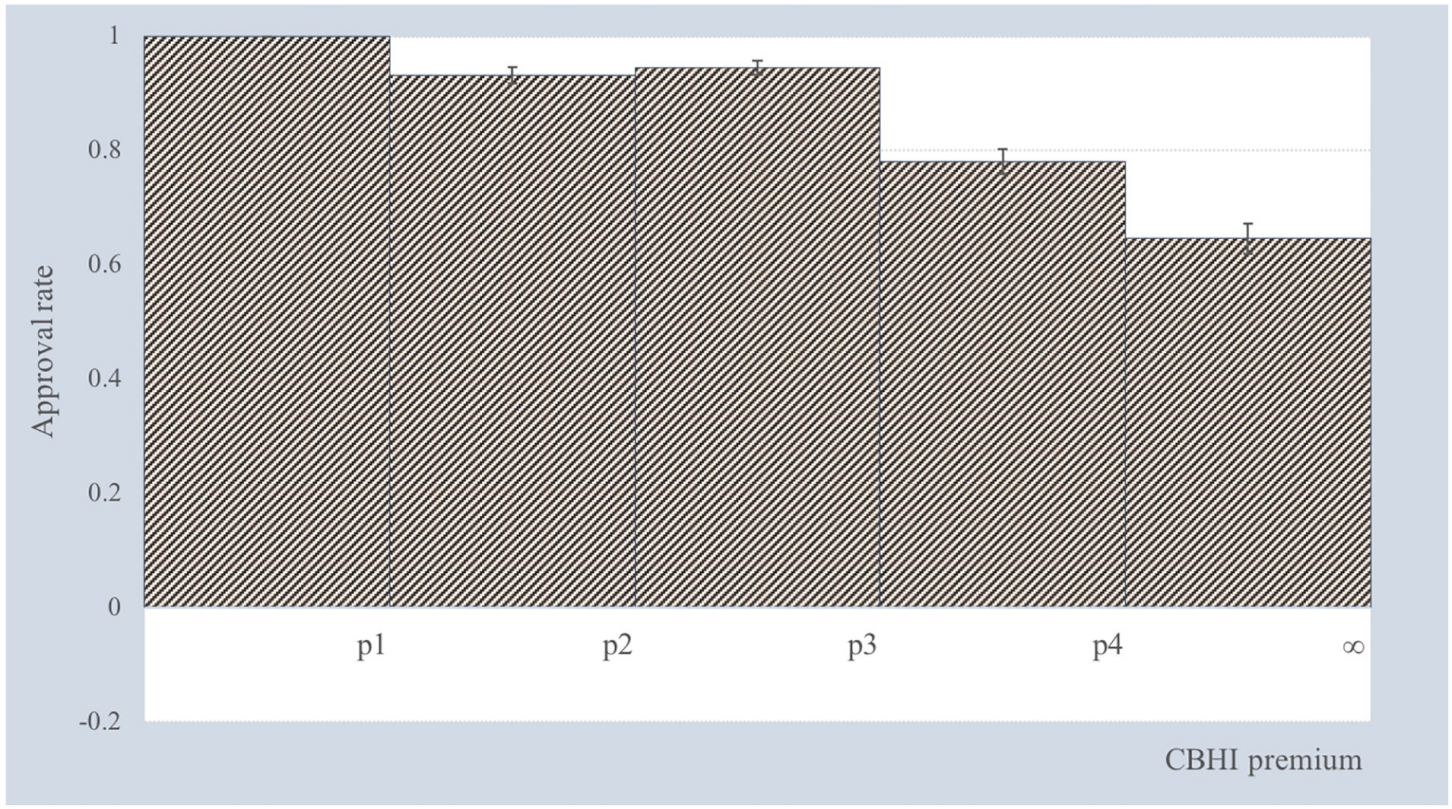
Approval rate for a hypothetical CBHI scheme.

According to the estimated marginal share of WTP, we measure the approval rate of the respondents for each CBHI premium interval as shown in Fig 3. The error bars display the 95% confidence intervals. The results show that 93.26% of the respondents will enroll in the CBHI scheme if the premium is between *p*_2_ and *p*_3_. While Fig 3 provides information on the distribution of the approval rate averaged across the possible levels of all attributes, the following section reports the distribution of the approval rate when individual attributes change from baseline level to new level.

**Fig 4:**
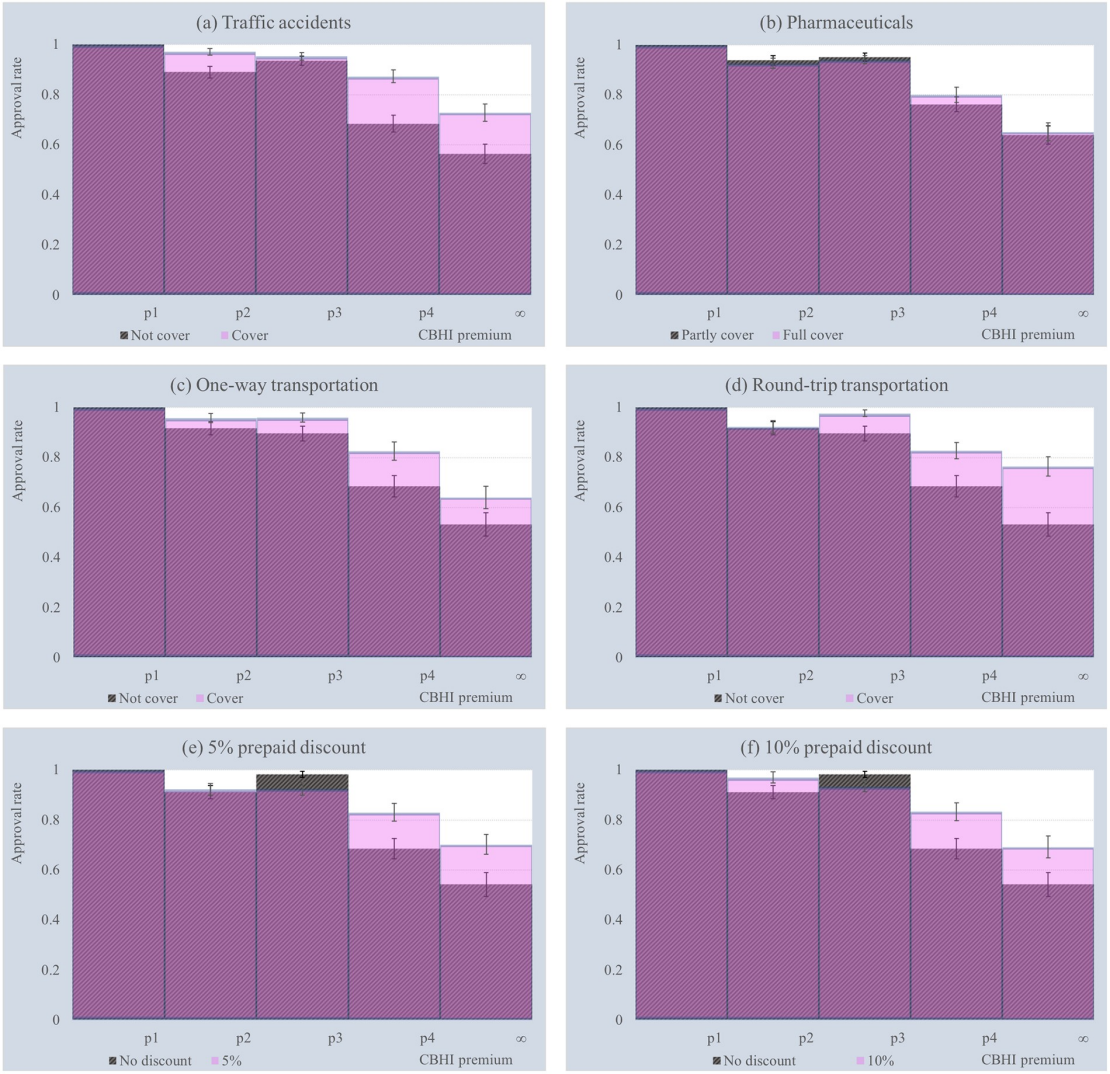
Approval rate by attribute.

As only hypothetical attributes are of interest, Table 2 shows the changes from the baseline level to the new level for each hypothetical attribute. The distribution of approval rates by attribute is presented in Fig 4, and the error bars show 95% confidence intervals. It is notable that the approval rate of the upper bins is significantly affected by the changes in level across all hypothetical attributes. In particular, the change in attribute levels increases the share of those whose WTP value is greater than *p*_4_.

**Table 2:** Attribute change from the baseline level to the new level.

	Baseline level (*a*_0_)	New level (*a*_1_)
(**a**)	Not cover traffic accidents	Cover traffic accidents
(**b**)	Partly cover pharmaceuticals	Fully cover pharmaceuticals
(**c**)	Not cover transportation	Cover one-way transportation
(**d**)	Not cover transportation	Cover round-trip transportation
(**e**)	No prepaid discount	5% prepaid discount
(**f**)	No prepaid discount	10% prepaid discount

Strikingly, compared to no insurance coverage for transportation, addressing the insurance coverage for round-trip transportation significantly increases the approval rate for the CBHI scheme with a price of more than *p*_4_ by 23.14 percentage points. The empirical evidence provided by this study supports the findings of [33] in rural Malawi and [16] in northwest Cambodia. These two studies employed a DCE approach to elicit SP on CBHI enrollment and found that greater transportation insurance coverage significantly influences respondents’ choice behavior. However, the findings of the present study are causally interpretable. The results indicate that transportation is a crucial component of CBHI scheme promotion in rural areas of Savannakhet Province.

Similar to our sample, a substantial number of people in remote areas of Lao PDR are highly dependent on public transportation to commute to health care facilities. However, until very recently, the limited public transportation services along with poor road conditions are also a fundamental source of excessive travel expenses. Hence, our findings provide useful insight for policy improvements to the CBHI scheme across Lao PDR considering the transportation barrier. In particular, the implementation of a CBHI scheme with insurance coverage for round-trip transportation might not only be more popular but might also increase the WTP among the targeted population in remote areas. Moreover, as a complement to redesigning the benefit package such that it fulfills the expectations of the targeted enrollees, developing the transportation infrastructure can serve as a supporting mechanism to ensure greater coverage of the CBHI scheme in Lao PDR.

## Conclusions

The goal of the CBHI scheme is to protect people against direct OOP payments and enhance their access to primary health care services by promoting enrollment, which is consistent with UHC. However, in many LMICs, the levels of enrollment in the implemented schemes remain far from satisfactory. The ordinary CBHI schemes cannot attract many people by their functional nature. Therefore, to make CBHI schemes more attractive, this study identifies SP and the WTP distribution for CBHI plans based on a set of hypothetical extended attributes—premium levels; a prepaid discount; and insurance coverage for medical consultations, hospitalizations, traffic accidents, pharmaceuticals, and transportation—in rural Lao PDR. We apply conjoint analysis using a fully randomized experimental design to elicit SP data because the estimates can be interpreted as a causal inference, and we apply a nonparametric WTP distribution analysis to measure the WTP distribution [36, 41].

The findings show that the composition of the benefit package has a crucial impact on respondents’ probability of enrolling in the CBHI scheme. In particular, the respondents value a hypothetical alternative policy over the status quo. The lower bound of the average WTP is estimated at 25,579 LAK per month, which is at least as much as 10.9% of the per capita income in the area (or 3.29% of the median household income). The average WTP found in this study is higher than the average WTP reported in the systematic review on WTP for health insurance in LMICs [29]. More importantly, the existence of round-trip transportation fee coverage significantly increases enrollment probabilities and WTP. In conclusion, low enrollment in the CBHI scheme in Lao PDR does not necessarily indicate low demand by potential enrollees. The enrollment rate can increase if the benefit package is improved and transportation factors are addressed.

An important limitation of this study is that we fail to conduct group discussions with sample respondents to obtain the most relevant attributes of scheme enrollment; this area merits future study.

## Supporting information

S1 Appendix Conjoint experiment sheet. (PDF)

S2 Appendix Questionnaire sheet in English. (PDF)

S3 Appendix Questionnaire sheet in Laotian. (PDF)
