# Supplementary material for: Potential demand for voluntary community-based health insurance improvement in rural Lao People’s Democratic Republic: A randomized conjoint experiment
Source: PLoS One. 2019 Jan 8;14(1):e0210355. doi: 10.1371/journal.pone.0210355 (PMC6324784; doi:10.1371/journal.pone.0210355)
Supplement: S1 Appendix — (PDF) [file pone.0210355.s001.pdf]

Supporting Information

1. Conjoint experiment

(Before the experiment, investigators have to read out loud the following information to each respondent and confirm the respondent's understanding at the end of the message.)

We aim to promote the improved health of self-employed households through CBHI enrollment expansion, which is a risk-pooling system, at the district level.

Below is a scenario presented before you; rank the policies that you think will maximize the benefit of the policy intervention.

"We would like to propose various policies for CBHI scheme improvement. We assume that the benefit packages in the hypothetical CBHI scheme cover out- and in-patient services. Under the CBHI scheme, healthcare would be first delivered by the contracting facilities (dispensaries and district hospitals) in your local area. Only referred patients are sent to provincial or regional hospitals. The premium can be paid monthly or annually. The window period of service access is three months upon enrollment.

We further assume that if every district achieves greater than or equal to 500 CBHI members, the quality of health care will gradually improve because district hospitals can improve cost recovery".

In the experiment, you are presented with five different choice tasks. Each choice task has three options: two hypothetical CBHI schemes, A and B, and the CBHI status quo scheme.

Each alternative is characterized by random levels of seven attributes, namely, *premium*; *insurance coverage for hospitalizations*, *medical consultations*, *traffic accidents*, *transportation*; and *prepaid discount*.

The levels of each attribute are demonstrated by the following images.

**1.1**	1 = 12,000<br>2-4 = 20,000<br>5-7 = 25,000<br>≥8 = 28,000<br>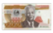<br>- 2,000	Premium per household per month is 2,000LAK cheaper than the current premium, i.g., 12,000LAK → 10,000LAK.
**1.2**	1 = 12,000<br>2-4 = 20,000<br>5-7 = 25,000<br>≥8 = 28,000<br>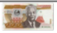	Current premium per household per month.
**1.3**	1 = 12,000<br>2-4 = 20,000<br>5-7 = 25,000<br>≥8 = 28,000<br>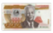<br>+ 2,000	Premium per household per month is 2,000LAK more expensive than the current premium, i.g., 12,000LAK → 14,000LAK.
**1.4**	1 = 12,000<br>2-4 = 20,000<br>5-7 = 25,000<br>≥8 = 28,000<br>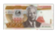<br>+ 4,000	Premium per household per month is 4,000LAK more expensive than the current premium, i.g., 12,000LAK → 16,000LAK.
**2.2**	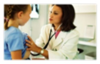	Coverage of the fee of technical examinations and disease diagnosis.
**3.2**	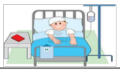	Coverage of the hospital bed costs if you stay overnight in the hospital (in which CBHI scheme has defined).
**4.2**	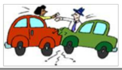	Coverage of charges of medical treatment due to traffic accident.
**5.1**	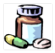	Coverage of only the pharmaceuticals that are identified in the essential medicines list defined by the Ministry of Health for each hospital level.
**5.2**	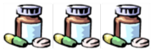	Coverage of all pharmaceuticals used for treatment.
**6.2**	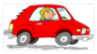	Coverage of one-way travel cost of the patient to a hospital out of the district.
**6.3**	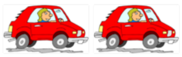	Coverage of round-trip travel cost of the patient to a hospital out of the district.
**7.2**	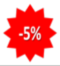	5% off for members who pay the CBHI premium fee 1 year in advance.
**7.3**	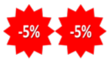	10% off for members who pay the CBHI premium fee 1 year in advance.

You are then asked to rank the three options in each choice task based on your preferences.

1= most preferred

2= average preferred

3= less preferred

Choice task 1:

	**Option A**	**Option B**	**Status quo**
**Premium**	1 = 12,000<br>2-4 = 20,000<br>5-7 = 25,000<br>≥8 = 28,000 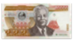 - 2,000	1 = 12,000<br>2-4 = 20,000<br>5-7 = 25,000<br>≥8 = 28,000 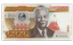 + 2,000	1 = 12,000<br>2-4 = 20,000<br>5-7 = 25,000<br>≥8 = 28,000 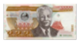
**Prepaid discount**		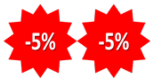	
**Hospitalizations**	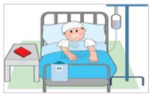		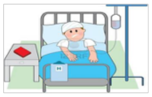
**Medical consultations**		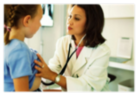	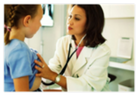
**Pharmaceuticals**	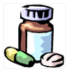	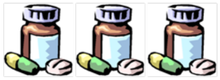	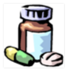
**Transportation**	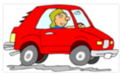	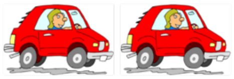	
**Traffic accidents**	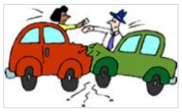	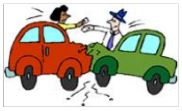	
	☐	☐	☐

(The respondent has to rank four additional choice tasks with different combinations of seven attribute levels)
