# Supplementary material for: Potential demand for voluntary community-based health insurance improvement in rural Lao People’s Democratic Republic: A randomized conjoint experiment
Source: PLoS One. 2019 Jan 8;14(1):e0210355. doi: 10.1371/journal.pone.0210355 (PMC6324784; doi:10.1371/journal.pone.0210355)
Supplement: S2 Appendix — (PDF) [file pone.0210355.s002.pdf]

General Instructions for Household Survey

Hello, my name is ………………………………………….. I am the interviewer in your household. This survey is under the supervision of Associate Professor GOTO Daisaku, Hiroshima University, Japan.

Title: Low Enrollment of Community-based Health Insurance Scheme: A Case Study of Rural Households in Savannakhet Province, Lao People's Democratic Republic

Objectives:

1. To examine the causal effects of benefit package components of hypothetical CBHI schemes on enrollment probabilities.
2. To elicit willingness to pay for CBHI scheme improvement.

Targets:

Increasing member district
Decreasing member district

Respondents:

CBHI members
Non-members
Ex-members

Study areas:

8 villages, Champhone and Xaibouly Districts, Savannket Province

In this survey, you will be asked about your household's demographic and socio-economic information and you will be involved in a conjoint experiment. All survey responses you provide for this study will be used only for academic purposes and will be kept completely confidential. Are you willing to participate in this study?

☐ Yes, I am ☐ No, I am not

Household survey on the preferences

on Community-based Health Insurance in Savannakhet province

Lao PDR (September 2016)

****

Household Questionaire

Household number: [ ]

Name of respondent: ……………………..…………………...…………………………..

Numbers of families living in the same household: [ ]

Household status for CBHI scheme:
1= member
2= ex-member
3= never

Did the household head work for the last 7 days?
1= Yes
2= No

Respondent status:
1= household chief
2= spous
3= children
4= another member

Main language:
1= Lao
2= Phutai
3= Hmong
4= other

Religion:
1= Buddism
2= Christian
3= Ghost
4= other

Nationality:
1= Lao
2= Vietnamese
3= Chinese
4= other

Ethnicity:
1= Lao lum
2= Phuthai
3= other

Contact number: ……...……………………………………………………………..……

Village name: ………………………………………………………………………..……

District: …………..……..…………………….……………………...……….…..…………

Longitude: ……..…………….………………………Latitude: ..……………….……………………

Date and time: [/ / 2016, :]

Investigator: …………………...……………….…………………………………...………

SECTION 1: Household roster

Please tell us the members of your household. Members are those who have been listed in the same family book. For household head's parents and spouse's parents that passed away, please tell us in Q9 - Q12, Q14, Q15.

1	2	3	4	5	6	7	8			9	10	11	12	13	14	15
**Person ID**	**Name**	**Relation to HH head**	**Sex**	**Age**	**Marital status**	**Habitation**	**Main occupation**			**Literate**	**Schooling years**	**Handicap**	**Chronic disease**	**Resent disease**	**Smoke**	**Drink alcohol**
		1 = head chief<br>2 = spouse<br>3 = children<br>4 = uncle/aunt<br>5 = parents<br>6 = bro./sis.<br>7 = …....in law<br>8 = other	1=M<br>2=F		1=single<br>2=married<br>3=divorced<br>4=widowed	1=permanent<br>2=temporal<br>3=visitor	*3 main occupations in the past 12 months*			1 = yes<br>0 = no	*exclude repeated classes*	*suffer from an handicap*<br>1 = yes<br>0 = no	*suffer from chronic disease*<br>1 = yes<br>0 = no	*have disease, accident, injury, etc during the last 3 months*<br>1 = yes<br>0 = no	*every day or at least twice a week*<br>1 = yes<br>0 = no	*every day or at least two days a week*<br>1 = yes<br>0 = no
							1	2	3							
**1**																
**2**																
**3**																
**4**																
**5**																
**6**																
**7**																
**8**																
**9**																
**10**																
**11**	hh head's father	5	1													
**12**	hh head's mother	5	2													
**13**	spouse's father	5	1													
**14**	spouse's mother	5	2													

Q 8. Main occupation:

Agriculture
- 1.1 = on own land
- 1.2 = tenant farmer
- 2 = animal husbandry
- 3 = fishing, hunting, NTFP
- 4 = HH enterprise
- 5 = craft

Salary employee
- 6.1 = gov't employee
- 6.2 = agric. sector
- 6.3 = non-agric. sector

Wage laborer
- 7.1 = agric. sector
- 7.2 = non-agric. sector

8 = money lending

Not income earners
- 9 = preschool (0-6 yrs old)
- 10 = student
- 11 = seeking for work
- 12 = housewife
- 13 = retired person
- 14 = unable to work due to disability
- 15 = others

SECTION 2: Living conditions

Pleae tell us about your living conidtions.

	Questions	Answers				
				Wall	Roof	Floor
1	Your house is mainly constructed by………	1<br>2<br>3<br>4<br>5<br>6	=<br>=<br>=<br>=<br>=<br>=	straw<br>mud<br>brick<br>wood<br>cement<br>other	= straw<br>= zinc<br>= tile<br>= wood<br>= other	=floor tile<br>=cement<br>= mud<br>=bamboo<br>= wood<br>= other
2	Do you have toilet in your household?	1<br>0	=<br>=	yes<br>no		
3	What is the main source of drinking water in your household?	1<br>2<br>3<br>4<br>5<br>6	=<br>=<br>=<br>=<br>=<br>=	buy bottle water<br>piped water<br>well / borehole<br>river / dam / lake<br>rain water from tank<br>other …….. ………………		
4	What is the main source of use water in your household?	1<br>2<br>3<br>4<br>5<br>6	=<br>=<br>=<br>=<br>=<br>=	buy bottle water<br>piped water<br>well / borehole<br>river / dam / lake<br>rain water from tank<br>other …….. ………………		
5	What is the main mode of lighting used in your household?	1<br>2<br>3<br>4<br>5<br>6	=<br>=<br>=<br>=<br>=<br>=	electricity<br>gas<br>oil<br>candle<br>wood<br>other …….. ………………		
6	What type of energy are you using mainly for cooking?	1<br>2<br>3<br>4<br>5	=<br>=<br>=<br>=<br>=	electricity<br>gas<br>oil<br>wood / charcoal<br>other …….. ………………		

SECTION 3: Farm land, production assets and animals, durable goods

Pleas tell us about your household's agricultural land and assets, and durable goods.

	Questions	Answers		
1	Please tell us about your household's agriuclulture land areas	m^2	1 =	land owned (purchased)
			2 =	land owned (inherited)
			3 =	land rented from others
			4 =	land rented out to others
			5 =	fallow land
			6 =	other land
2	Please tell us about your household's production assets	Quantity	1 =	tractors owned
			2 =	tractors shared with other hhs
			3 =	trucks
			4 =	yanma
			5 =	threshing machine
			6 =	rice mill
3	How many for each of these animals does your family own?	Quantity	1 =	buffalo
			2 =	cow
			3 =	pigs
			4 =	goat
			5 =	poultry
4	In your household, is there…………..?	Quantity	1 =	bicycle
			2 =	motorbike
			3 =	car
5	In your household, how many ……….. are there? Which are still working now?	Quantity	1 =	TV
			2 =	telephone
			3 =	fridge
			4 =	electric pan
			5 =	electric water boiling pot
			6 =	washing machine
			7 =	frozen machine
			8 =	gas stove
			9 =	fans

SECTION 4: Income

Please tell us your household income earning in the past 12 months

	Income source	1 year production	Sale	Unit price	Total sale amount	other cost (labor, fertilizer)
1	rice	kg	kg	kip	kip	kip

	Income source	1 year sale	other cost (labor, fertilizer, animal feed)
2	vegetables	kip	kip
3	fruits	kip	kip
4	animals	kip	kip
5	handicraft	kip	kip
6	rubber plantation	kip	kip
7	sugarcane	kip	kip
8	Eucalyptus	kip	kip
9	cassava	kip	kip
10	other	kip	kip

	Income source	1 year sale
11	fishery	kip
12	NTFP	kip
13	charcoal	kip
14	other ……………….	kip

	Income source	1 year earn
15	wage	kip
16	salary	kip
17	deposit interest	kip
18	HH enterprise profit	kip
19	remittance	kip
20	renting	kip
21	pension	kip
22	other………………	kip

SECTION 5: Expenditure

How much does your household pay for ……….a month or a year? *Remark: you can answer amount spend either in a month or in a year.*

	Expenditure source	Amount spent (kip)	
		1 month	1 year
1	tranaportation		
2	education		
3	enery (ele, gas, wood, charcoal, oil, etc)		
4	water		
5	telephone		
6	cloth, hair, cosmetics		
7	health care		
8	food		
9	money transfer		
10	rent		
11	weddings		
12	cash donation to temples		
13	associations fee		
14	fixing		
15	investment in business (purchase animals)		
16	other……………………..…………		

SECTION 6: Borrowing

Please tell us about your household's borrowig experience which is still active up to now and already finished

	Borrowing source<br>1 = bank<br>2 = financial institution<br>3 = microfinance<br>4 = associations<br>5 = insurer<br>6 = village fund<br>7 = fiends/cousins<br>8 = other	mm/yy of borrowing	Borrowing amount	Purpose of borrowing?<br>1 = agriculture<br>2 = animal raising<br>3 = health care<br>4 = education<br>5 = daily expense<br>5 = non-agric. investment<br>7 = other	Annual interest rate (%)	Payment due	Owing amount
1	2	3	4	5	6	7	8
1							
2							
3							

SECTION 7: Deposit

Please tell us about your household's deposit experience which is still active up to now

	Name of financial institution<br>1 = bank<br>2 = financial institution<br>3 = microfinance<br>4 = associations<br>5 = insurer<br>6 = fiends/cousins<br>7 = other	mm/yy of deposit	Deposit amount	Annual interest rate (%)
1	2	3	4	5
1				
2				
3				

SECTION 8: Saving in kind

Please tell us about your household's saving in kind.

	Item	Amount (kip)
1	2	3
1	gold	
2	jelwery	

SECTION 9: Community-based Health Insurance (CBHI)

Please tell us about your CBHI information

	Questions	Answers
1	What is your household's status in the CBHI?	1 = current member (go to #4)<br>2 = ex-member (go to #3)<br>3 = never
2	Have you ever heard of CBHI?	1 = yes<br>0 = no (go to Section 10)
3	Why have you never applied for CBHI?	Rank 1-3<br>☐ 1 = not enough information<br>☐ 2 = too expensive fee<br>☐ 3 = not enough financial sources<br>☐ 4 = poor services of contracted hospitals<br>☐ 5 = no trust management of CBHI<br>☐ 6 = not trust beneficiaries<br>☐ 7 = other…………………………
4	How do / did you know about CBHI scheme?	Rank 1-3<br>☐ 1 = from a family member<br>☐ 2 = from a friend<br>☐ 3 = from the care provider<br>☐ 4 = from the staff of that health insurance<br>☐ 5 = from association<br>☐ 6 = from a community leader<br>☐ 7 = from media<br>☐ 8 = from other member of health insurance<br>☐ 9 = other…………………………
5	Have you ever droped out and renewed the membership?	1 = yes<br>0 = no (go to #7)
6	If yes, please tell us the membership details in each period	From ……………..……To……………….<br>From ……………..……To……………….
7	Which year are / were you member?	……………………….
8	Which year did you drop out?	……………………….
9	Do / did you pay the contribution regularly to the CBHI office?	1 = yes (go to #11)<br>0 = no
10	Why do you not pay regularly?<br><br>*Ex-member:* why have you stopped paying the fee?	Rank 1-3<br>☐ 1 = no collector<br>☐ 2 = too expensive cost<br>☐ 3 = not enough financial sources<br>☐ 4 = poor services<br>☐ 5 = no trust management of CBHI<br>☐ 6 = not trust beneficiaries<br>☐ 7 = other…………………………

	Questions	Answers
11	Do you know the managers of CBHI in village and district levels?	Yes No<br>1 = 0 chief executive / chairman<br>1 = 0 other staff of CBHI
12	How many members of CBHI do you know in this village?	……………………….. persons
13	What do you think are the benefits for being CBHI member?	Rank 1-3<br>☐ 1= more easily access to health service<br>☐ 2= health care cost reduction<br>☐ 3= important for emergency case in the future<br>☐ 4= better health condition<br>☐ 5= other (specify) ………………………..

SECTION 10: Health and Access to Care

Please tell us about your and family members' health and health care access

	Questions	Answers
1	How far from your house is the nearest health facility?	………………… Km
2	What transportation mode do you use to get to the health facilities?	1 = on foot<br>2 = motorbike<br>3 = car<br>4 = hire vehicle of others<br>5 = other (specify) ………………
3	Are there any household members who was hospitalized over the last two years?	No Yes<br>0 1 if yes, ………………persons
4	Over the past two years, are there any members in your family who you think should be hospitalized but not due to lack of money?	No Yes<br>0 1 if yes, ………………persons

5 : *Please specify those who received the health care during the last 12 months*

For member and ex-member after 9/2015 (applies to the last two columns)

Person ID	Type of health care seeking<br><br>1 = hospitalization<br>2 = outpatient care<br>3 = tradditional care	Amount paid for service?<br><br>(kip)	Amount paid for medicine<br><br>(kip)	Amount paid for other cost<br><br>(kip)<br><br>*e.g. acoompany stay, transporation, meal…*	Where did you take money from?<br><br>1 = income<br>2 = saving<br>3 = borrowing<br>4 = sale<br>5 = other	Does CBHI cover health care cost?<br><br>*service + medicine cost*<br><br>0 = no<br>1 = partly covered<br>2 = total covered	Can you evaluate the service received from CBHI insurance?<br><br>1 = very satisfied<br>2 = satisfied<br>3 = average<br>4 = bad<br>5 = very bad
**1**							
**2**							
**3**							
**4**							
**5**							
**6**							
**7**							
**8**							
**9**							
**10**							

SECTION 11: Social Networks

Please tell us about your and family members' social networks

	Questions	Answers
1	How long do you (hh head) live in the village?	…………………………..years
2	Apart from CBHI, what kinds of associations are family members a member?	1 = village party<br>2 = village authority<br>3 = women union<br>4 = youth union<br>5 = labor union<br>6 = neo home<br>7 = village police<br>8 = village soldier<br>9 = education association<br>10 = animal husbandry cooperative<br>11 = rice plantation cooperative<br>12 = village fund<br>13 = microfinance fund<br>14 = sport association<br>15 = NGO<br>16 = other….. ……………………
3	If a community project does not directly benefit you, but has benefits for many others in the village, would you contribute time or money to this project?	Yes No<br>1 0 time<br>1 0 money
4	In general, where do your family members always receive information about politics, government projects, associations, projects or the community?	Rank 1 - 3<br>☐ 1 = relatives, friends, neighbors<br>☐ 2 = village office board<br>☐ 3 = local market<br>☐ 4 = radio<br>☐ 5 = television<br>☐ 6 = groups or associations<br>☐ 7 = community leaders<br>☐ 8 = an agent of the government<br>☐ 9 = by NGOs<br>☐ 10 = other (specify)
5	Have you ever lent your neighbor during the past 12 months?	1 = yes (………… times)<br>0 = no
6	How much did you lend them per time?	…………………………..kip
7	How far is your house to the nearest local market?	…………………………..km

SECTION 12: Shocks

Please tell us about the following shocks occurred since 2012.

Types of shocks	Period of shocks				How much did you lose / pay for it?
	From		To		
	mm	yy	mm	yy	(kip)
**1. Death**<br>a. relationship:………………………………<br>b. age: ……………………………………..<br>c. occupation:………………………………					
**2. Accident**<br>a. who?:……………………………..……<br>b. what accident?: …………………...……..					
**3. Anthrax diesease in animals**<br>1. kind of animal:..…………………………..……<br># of damaged animal: ……….……...….. 2. kind of animal:..…………………………..……<br># of damaged animal: ……….……...…..<br>3. kind of animal:..…………………………..……<br># of damaged animal: ……….…...……..					
**4. Crop failure due to natural disaster and pests**<br>1. kind of crop:....…………………………..……<br># of damaged crop: ...……….……...…..<br>2. kind of crop:....…………………………..……<br># of damaged crop: ...……….……...…..<br>3. kind of crop:....…………………………..…<br># of damaged crop: ...……….……...…..					
**5. Disaster**<br>a. what disaster?:..…………………………..……<br>b. how long does it last: ...……………..……..					
**6. Robbery**<br>a. kinds robbed:..…………………………..……					
**7. Weddings ceremony:** ……....…… times					
**8. Sell:** land, house, bike, car, ...					
**9. Buy:** land, house, bike, car, yanma, rice mill, …					
**10. Other** ……………………………...………..					

SECTION 13: Conjoint Experiment

(Before the experiment, investigators have to read out loud the following information to each respondent and confirm the respondent's understanding at the end of the message.)

We aim to promote the improved health of self-employed households through CBHI enrollment expansion, which is a risk-pooling system, at the district level.

Below is a scenario presented before you rank the policies that you think will maximize the benefit of the policy intervention.

"We would like to propose various policies for CBHI scheme improvement. We assume that the benefit packages in the hypothetical CBHI scheme cover out- and in-patient services. Under the CBHI scheme, healthcare would be first delivered by the contracting facilities (dispensaries and district hospitals) in your local area. Only referred patients are sent to provincial or regional hospitals. The premium can be paid monthly or annually. The window period of service access is three months upon enrollment.
We further assume that if every district achieves greater than or equal to 500 CBHI members, the quality of health care will gradually improve because district hospitals can improve cost recovery".

In the experiment, you are presented with five different choice tasks. Each choice task has three options: two hypothetical CBHI schemes, A and B, and the CBHI status quo scheme.
Each alternative is characterized by random levels of seven attributes, namely, *premium; insurance coverage for hospitalizations, medical consultations, pharmaceuticals, traffic accidents, transportation; and prepaid discount.*

The levels of each attribute are demonstrated by the following images.

1.1	1 = 12,000<br>2-4 = 20,000<br>5-7 = 25,000<br>≥8 = 28,000<br>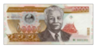<br>- 2,000	Premium per household per month is 2,000LAK cheaper than the current premium, i.g., 12,000 kip → 10,000 kip.
1.2	1 = 12,000<br>2-4 = 20,000<br>5-7 = 25,000<br>≥8 = 28,000<br>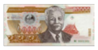	Current premium per household per month.
1.3	1 = 12,000<br>2-4 = 20,000<br>5-7 = 25,000<br>≥8 = 28,000<br>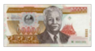<br>+ 2,000	Premium per household per month is 2,000LAK more expensive than the current premium, i.g., 12,000 kip → 14,000 kip.
1.4	1 = 12,000<br>2-4 = 20,000<br>5-7 = 25,000<br>≥8 = 28,000<br>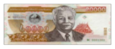<br>+ 4,000	Premium per household per month is 4,000LAK more expensive than the current premium, i.g., 12,000 kip → 16,000 kip.
2.2	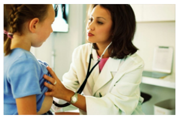	Cover the fee for technical examinations and disease diagnosis.
3.2	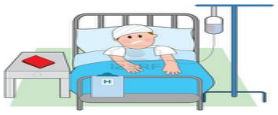	Cover the hospital bed costs if you stay overnight in the hospital (in which CBHI scheme has defined).
4.2	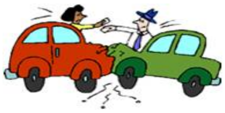	Cover charges of medical treatment due to traffic accident.
5.1	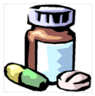	Cover only the pharmaceuticals that are mentioned in the essential medicines list defined the Ministry of Health for each level of hospital.
5.2	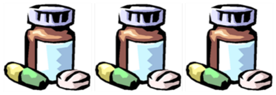	Cover all pharmaceutical charge used for the treatment.
6.2	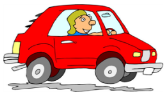	Cover one-way travel cost of the patient to a hospital out of the district.
6.3	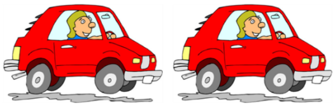	Cover round-ttrip travel cost of the patient to a hospital out of the district.
7.2	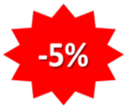	5% off for members who pay the CBHI premium fee 1 year in advance.
7.3	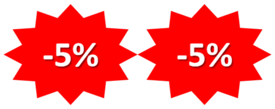	10% off for members who pay the CBHI premium fee 1 year in advance.

You are then asked to rank the three options in each choice task based on your preferences.

1= most preferred

2= average preferred

3= less preferred

<u>For example:</u>

	Option A	Option B	Status quo
**Premium**	1 = 12,000<br>2-4 = 20,000<br>5-7 = 25,000<br>≥8 = 28,000<br>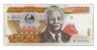<br>- 2,000	1 = 12,000<br>2-4 = 20,000<br>5-7 = 25,000<br>≥8 = 28,000<br>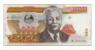<br>+ 2,000	1 = 12,000<br>2-4 = 20,000<br>5-7 = 25,000<br>≥8 = 28,000<br>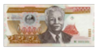
**Prepaid discount**		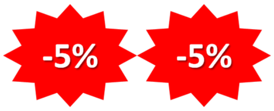	
**Hospitalizations**	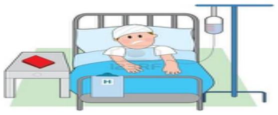		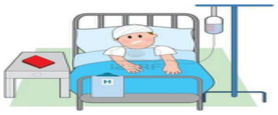
**Medical consultations**		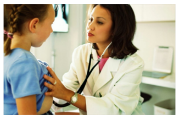	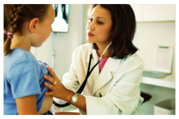
**Pharmaceuticals**	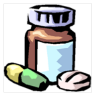	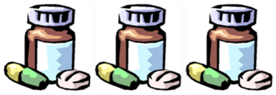	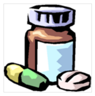
**Transportation**	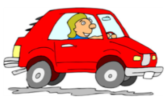	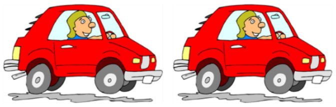	
**Traffic accidents**	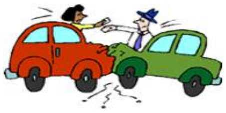	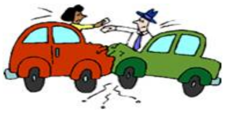	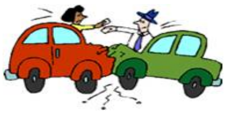
	1	3	2

Please rank the following 5 choice tasks based on your preferences.

<u>Choice set 75</u>

Choice task 1:

	Option A	Option B	Status quo
**Medical consultations**		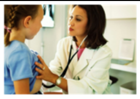	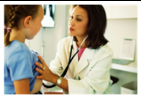
**Traffic accidents**		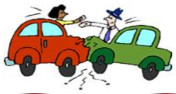	
**Transportation**		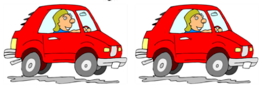	
**Pharmaceuticals**	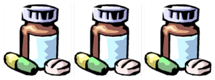	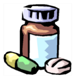	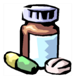
**Premium**	1 = 12,000<br>2-4 = 20,000<br>5-7 = 25,000<br>≥8 = 28,000 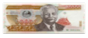 + 4,000	1 = 12,000<br>2-4 = 20,000<br>5-7 = 25,000<br>≥8 = 28,000 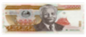 + 4,000	1 = 12,000<br>2-4 = 20,000<br>5-7 = 25,000<br>≥8 = 28,000 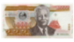
**Hospitalizations**	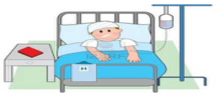	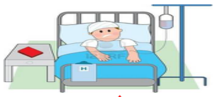	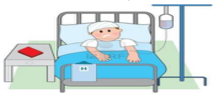
**Prepaid discount**		-5%	
	☐	☐	☐

Choice task 2:

	Option A	Option B	Status quo
**Medical consultations**		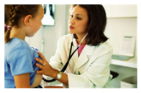	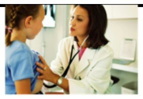
**Traffic accidents**		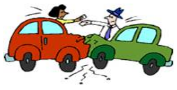	
**Transportation**		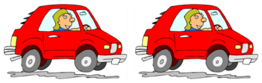	
**Pharmaceuticals**	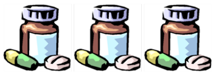	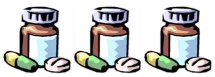	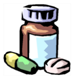
**Premium**	1 = 12,000<br>2-4 = 20,000<br>5-7 = 25,000<br>≥8 = 28,000 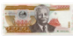	1 = 12,000<br>2-4 = 20,000<br>5-7 = 25,000<br>≥8 = 28,000 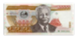 - 2,000	1 = 12,000<br>2-4 = 20,000<br>5-7 = 25,000<br>≥8 = 28,000 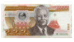
**Hospitalizations**			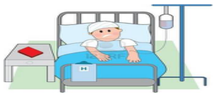
**Prepaid discount**	-5%	-5%	
	☐	☐	☐

Choice task 3:

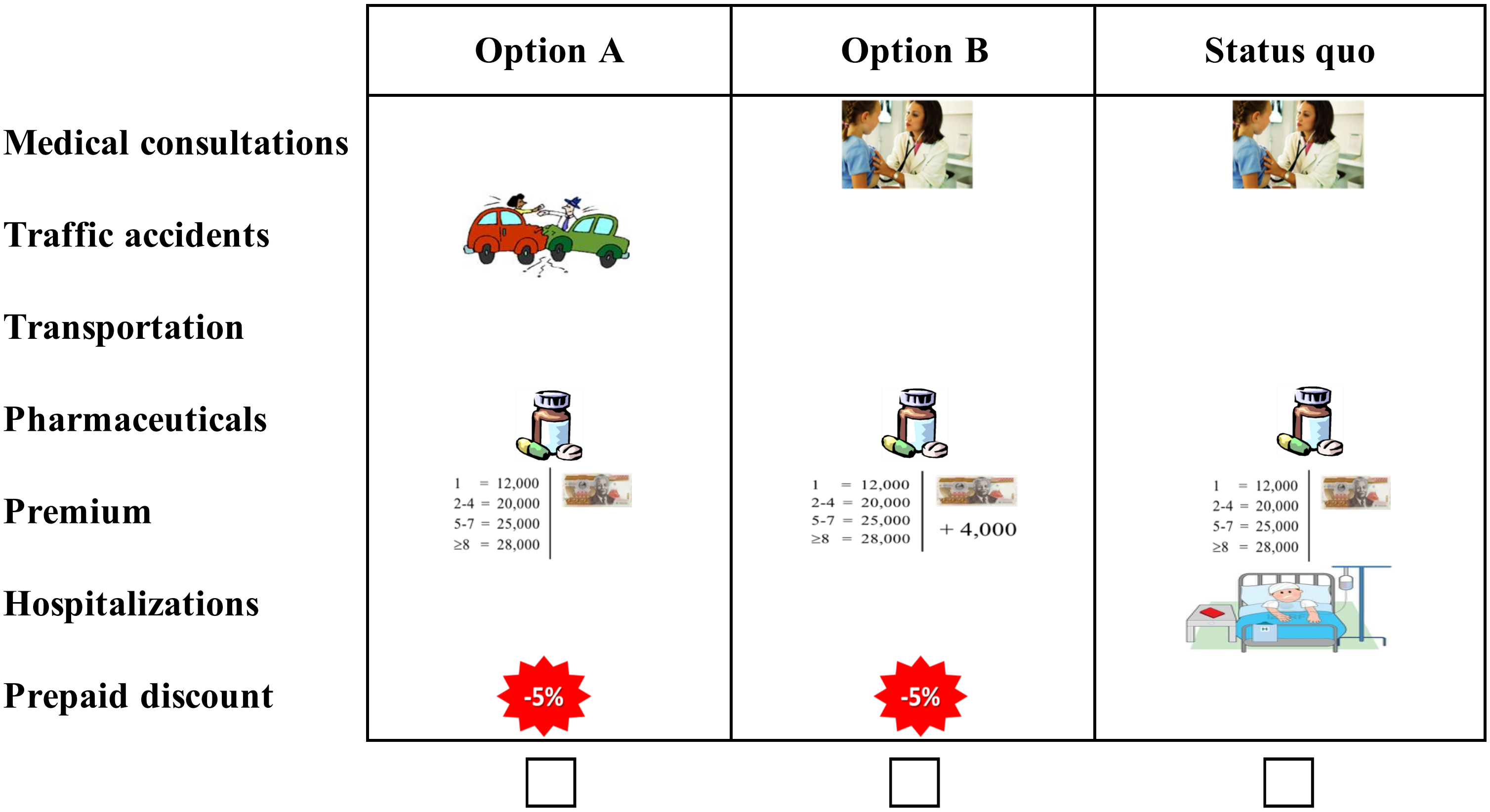

Choice task 4:

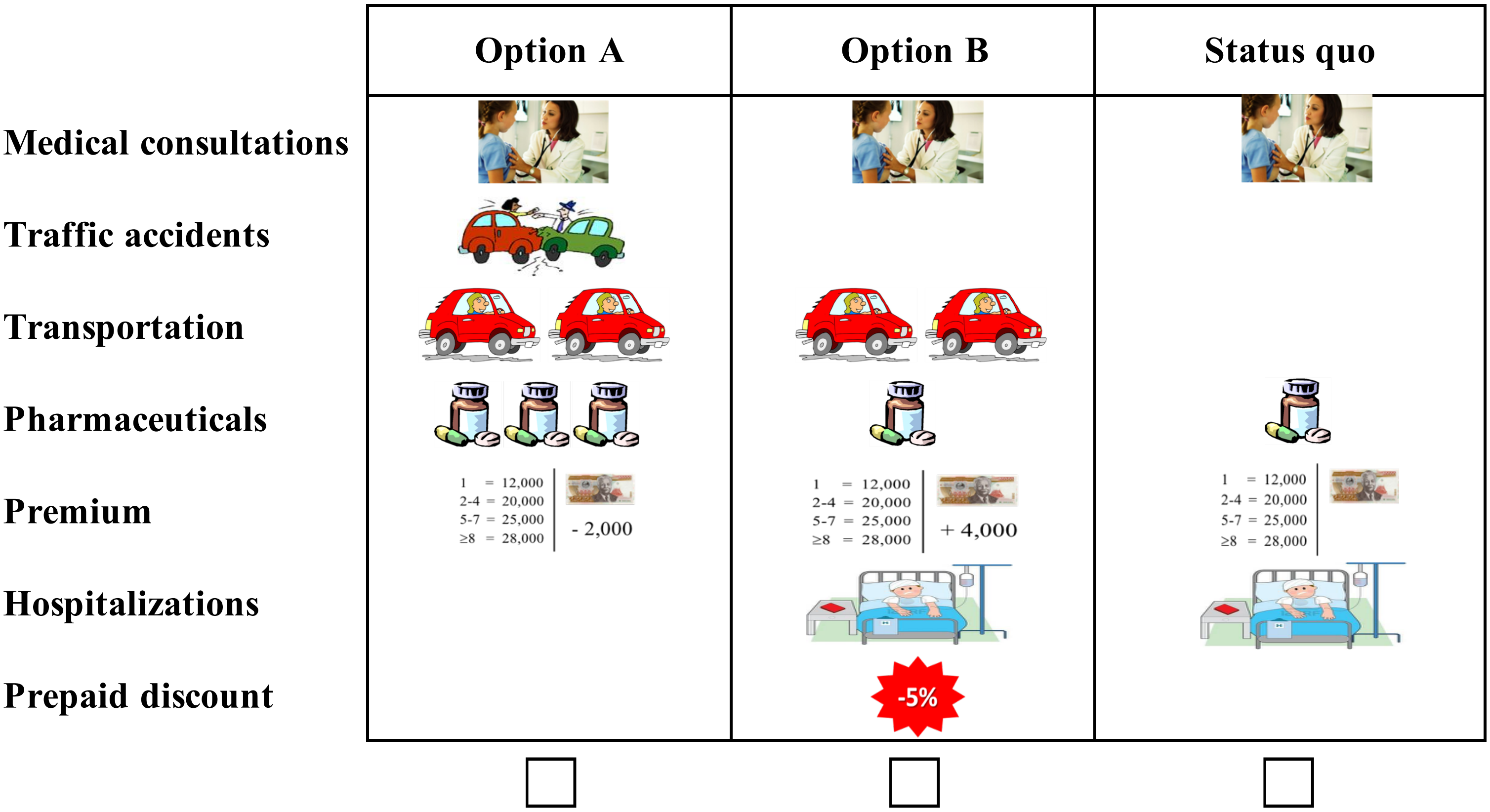

Choice task 5:

	Option A	Option B	Status quo
**Medical consultations**		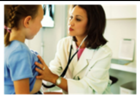	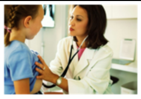
**Traffic accidents**			
**Transportation**		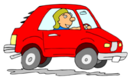	
**Pharmaceuticals**	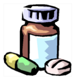	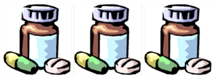	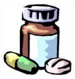
**Premium**	1 = 12,000<br>2-4 = 20,000<br>5-7 = 25,000<br>≥8 = 28,000<br>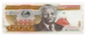<br>+ 4,000	1 = 12,000<br>2-4 = 20,000<br>5-7 = 25,000<br>≥8 = 28,000<br>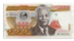<br>- 2,000	1 = 12,000<br>2-4 = 20,000<br>5-7 = 25,000<br>≥8 = 28,000<br>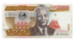
**Hospitalizations**	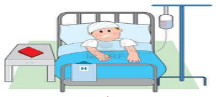	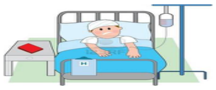	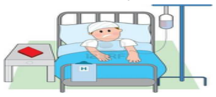
**Prepaid discount**	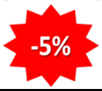	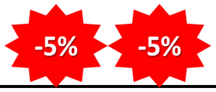	
	☐	☐	☐
