# Supplementary material for: Potential demand for voluntary community-based health insurance improvement in rural Lao People’s Democratic Republic: A randomized conjoint experiment
Source: PLoS One. 2019 Jan 8;14(1):e0210355. doi: 10.1371/journal.pone.0210355 (PMC6324784; doi:10.1371/journal.pone.0210355)
Supplement: S3 Appendix — (PDF) [file pone.0210355.s003.pdf]

ຄຳແນະນຳສຳລັບການສຳຫລວດຄົວເຮືອນກ່ຽວກັບລະບົບ ກປສຊ

ສະບາຍດີ, ຂ້າພະເຈົ້າຊື່ວ່າ: ………………………………………….. ເປັນຜູ້ສຳພາດຄົວເຮືອນຂອງທ່ານ. ການສຳຫຼວດໃນຄັ້ງນີ້ ແມ່ນໄດ້ຮັບການຊີ້ນຳຈາກ ຮອງສາດສະດາຈານ GOTO Daisaku, ມະຫາວິທະຍາໄລ ຮິໂຣຊິມາ, ປະເທດຍີປຸ່ນ.

<u>ຫົວຂໍ້:</u> ອັດຕາປົກຫຸ້ມຂອງລະບົບ ກປສຊ ທີ່ຍັງຕ່ຳ: ກໍລະນີສຶກສາ ຄົວເຮືອນ ຢູ່ແຂວງສະຫວັນນະເຂດ, ສປປ ລາວ.

<u>ຈຸດປະສົງ:</u>

1. ເພື່ອສຶກສາເບິ່ງຜົນກະທົບທີ່ເປັນສາເຫດຂອງອົງປະກອບຕ່າງໆ ກ່ຽວກັບການຄຸ້ມຄອງຂອງລະບົບ ກປສຊ ຕໍ່ກັບອັດຕາການເຂົ້າຮ່ວມຂອງຄົວເຮືອນ.
2. ເພື່ອສຶກສາເບິ່ງ ຄວາມພໍໃຈຈ່າຍຂອງຄົວເຮືອນ ເພື່ອການປັບປຸງລະບົບ ກປສຊ.

<u>ເປົ້າໝາຍ:</u>

ເມືອງທີ່ມີສະມາຊິກ ກອງທຶນ ກປສຊ ເພີ່ມຂຶ້ນຕໍ່ເນື່ອງ
ເມືອງທີ່ມີສະມາຊິກ ກອງທຶນ ກປສຊ ຫຼຸດລົງຕໍ່ເນື່ອງ

<u>ຜູ້ທີ່ຕອບແບບສອບຖາມ:</u>
ຜູ້ທີ່ເຂົ້າກອງທຶນ ກປສຊ
ຜູ້ທີ່ເປັນກຸ່ມເປົ້າໝາຍ ແຕ່ບໍ່ເຄີຍເຂົ້າກອງທຶນ ກປສຊ
ຜູ້ທີ່ເຄີຍເຂົ້າກອງທຶນ ກປສຊ ແຕ່ປະຈຸບັນຢຸດຕິແລ້ວ

<u>ເຂດສຶກສາ:</u>

8 ບ້ານ, 2 ເມືອງ: ຈຳພອນ ແລະ ໄຊບູລີ, ແຂວງສະຫວັນນະເຂດ

ໃນການສຳຫລວດຄັ້ງນີ້, ທີມງານສຳຫລວດ ຈະໄດ້ສຳພາດຂໍ້ມູນກ່ຽວກັບສະມາຊິກໃນຄົວເຮືອນຂອງທ່ານ ແລະ ຂໍ້ມູນທາງດ້ານເສດຖະກິດ-ສັງຄົມໃນຄົວເຮືອນຂອງທ່ານ ແລະ ທ່ານຍັງຈະໄດ້ເຂົ້າຮ່ວມເຮັດການທົດລອງຕື່ມອີກ. ຂໍ້ມູນຕ່າງໆຈາກການສຳຫລວດແມ່ນຖືກນຳໃຊ້ເພື່ອການສຶກສາເທົ່ານັ້ນ ແລະ ຈະຖືກຮັກສາເປັນຄວາມລັບ. ທ່ານຍິນຍອມທີ່ຈະໃຫ້ສຳພາດ ແລະ ຍິນດີທີ່ຈະເຂົ້າຮ່ວມການເຮັດທົດລອງບໍ່?

☐ ຍິນຍອມ ☐ ບໍ່ຍິນຍອມ

ການສຳຫລວດຄົວເຮືອນ ກ່ຽວກັບລະບົບ ກປສຊ

ແຂວງສະຫວັນນະເຂດ

ສປປ ລາວ (ກັນຍາ 2016)

****

ແບບສອບຖາມສຳລັບຄົວເຮືອນ

ເລກທີຄົວເຮືອນ: [ ]

ຊື່ຜູ້ຖືກສຳພາດ:……………………………………..…………………………

ຈຳນວນຄອບຄົວທີ່ອາໄສຢູ່ໃນເຮືອນຫຼັງດຽວກັນ: [ ]

ຄົວເຮືອນທ່ານເປັນສະມາຊິກກອງທຶນ ກປສຊບໍ່?

1= ສະມາຊິກ
2= ເຄີຍເປັນສະມາຊິກ
3= ບໍ່ແມ່ນສະມາຊິກ

1 ອາທິດຜ່ານມາ ຫົວໜ້າຄົວເຮືອນທ່ານ ໄດ້ເຮັດວຽກບໍ່?

1= ເຮັດວຽກ
2= ບໍ່ໄດ້ເຮັດວຽກ

ສາຍພົວພັນຂອງຜູ້ຖືກສຳພາດ:

1= ຫົວໜ້າຄົວເຮືອນ
2= ຜົວ ຫຼື ເມຍ ຂອງຫົວໜ້າຄົວເຮືອນ
3= ລູກ
4= ສະມາຊິກຄົວເຮືອນຄົນອື່ນໆ

ພາສາ:

1= ລາວ
2= ຜູ້ໄທ
3= ມົ້ງ
4= ອື່ນໆ

ສາດສະໜາ:

1= ພຸດ
2= ຄຣິດສະຕຽນ
3= ຜີ
4= ອື່ນໆ

ເຊື້ອຊາດ:

1= ລາວ
2= ຫວຽດນາມ
3= ຈີນ
4= ອື່ນໆ

ຊົນເຜົ່າ:

1= ລາວລຸ່ມ
2= ຜູ້ໄທ
3= ອື່ນໆ

ເບີໂທຕິດຕໍ່: ……………………..……………………………………………………………..

ຊື່ບ້ານ:……………..……………………………………………………………………..……

ຊື່ເມືອງ……………..………………………………..……………………..……..……………

ເສັ້ນສູນສູດ: ………………..………………………ເສັ້ນແວງ: ………………..……………………

ວັນທີ ແລະ ເວລາ: [/ / 2016, :]

ຊື່ຜູ້ສຳພາດ: ……………………..……………………………………………………..…………

ພາກທີ1: ຂໍ້ມູນກ່ຽວກັບສະມາຊິກຂອງຄົວເຮືອນ

ກະລຸນາບອກລາຍລະອຽດ ກ່ຽວກັບສະມາຊິກໃນຄົວເຮືອນຂອງທ່ານ ຜູ້ເຊິ່ງມີລາຍຊື່ຢູ່ໃນປື້ມສຳມະໂນຄົວດຽວກັນກັບທ່ານ. ສຳລັບ ພໍ່ແມ່ຂອງຫົວໜ້າຄອບຄົວ ແລະ ພໍ່ແມ່ຂອງຄູ່ສົມລົດຂອງຫົວໜ້າຄອບຄົວ ທີ່ເສຍຊີວິດແລ້ວ ແມ່ນໃຫ້ຕອບແຕ່ຂໍ້ Q9 - Q12, Q14, Q15.

1	2	3	4	5	6	7	8			9	10	11	12	13	14	15
**ລະຫັດບຸກຄົນ**	**ຊື່**	**ສາຍພົວພັນກັບຫົວໜ້າຄອບຄົວ**	**ເພດ**	**ອາຍຸ**	**ສະຖານະ**	**ທ່ານຢູ່ເຮືອນປະຈຳບໍ່?**	**ອາຊີບຕົ້ນຕໍ**			**ອ່ານໜັງສືອອກບໍ່?**	**ຈຳນວນປີການສຶກສາ**	**ພິການ**	**ພະຍາດຊຳເຮື້ອ**	**ພະຍາດທີ່ເປັນ 3 ເດືອນຜ່ານມາ**	**ສູບຢາ**	**ດື່ມເຫຼົ້າ**
		1 =ຫົວໜ້າຄົວເຮືອນ<br>2 =ຜົວ,ເມຍ<br>3 =ລູກ<br>4 =ປ້າ,ລຸງ,ອາວ,ອາ,ນ້າບ່າວ,ນ້າສາວ<br>5 = ພໍ່,ແມ່<br>6 =ອ້າຍ,ເອື້ອຍ,ນ້ອງ<br>7=ເຂີຍ,ໄພ້<br>8 =ອື່ນໆ	1=ຊາຍ<br>2=ຍິງ		1=ໂສດ<br>2=ແຕ່ງງານ<br>3=ຢ່າຮ້າງ<br>4=ໝ້າຍ	1=ປະຈຳ<br>2=ຊົ່ວຄາວ<br>3=ຢ້ຽມຢາມ	*ໃຫ້ທ່ານບອກອາຊີບຕົ້ນຕໍຂອງທ່ານ 3 ຢ່າງ ໃນໄລຍະ 12 ເດືອນຜ່ານມາ*				*ບໍ່ນັບເອົາຫ້ອງປີທີ່ຮຽນຊ້ຳຫ້ອງ*	*ທ່ານມີຄວາມພິການຂອງຮ່າງກາຍບໍ່*	*ທ່ານເປັນພະຍາດຊຳເຮື້ອ ປິ່ນປົວບໍ່ຫາຍບໍ່?*	*ເຊັ່ນ: ອຸປະຕິເຫດ, ຖືກບາດເຈັບ, ພະຍາດອື່ນໆ*	*ສູບທຸກວັນ ຫຼື ຢ່າງໜ້ອຍ 2 ຄັ້ງຕໍ່ອາທິດ*	*ສູບທຸກວັນ ຫຼື ຢ່າງໜ້ອຍ 2 ຄັ້ງຕໍ່ອາທິດ*
							1	2	3	1 =ໄດ້<br>0 =ບໍ່		1 =ມີ<br>0 =ບໍ່	1 =ມີ<br>0 =ບໍ່	1 =ມີ<br>0 =ບໍ່	1 =ສູບ<br>0 =ບໍ່	1 =ດື່ມ<br>0 =ບໍ່
1																
2																
3																
4																
5																
6																
7																
8																
9																
10																
11	ພໍ່ຂອງຫົວໜ້າ ຄຄ	5	1													
12	ແມ່ຂອງຫົວໜ້າ ຄຄ	5	2													
13	ພໍ່ຂອງຄູ່ສົມລົດ	5	1													
14	ແມ່ຂອງຄູ່ສົມລົດ	5	2													

Q 8. ອາຊີບ:

ກະສິກຳ:
1.1 =ໃນດິນຂອງຕົນເອງ
1.2 =ເຊົ່ານາຄົນອື່ນເຮັດ
2 =ລ້ຽງສັດ
3 = ຫາປາ, ລ່າສັດ, ຫາເຄື່ອງປ່າຂອງດົງ
4 =ຄ້າຂາຍ
5 =ຫັດຖະກຳ

ພະນັກງານ:
6.1 =ລັດຖະກອນ
6.2 =ຂົງເຂດກະສິກຳ
6.3 =ຂົງເຂດອື່ນໆ (ທີ່ບໍ່ແມ່ນກະສິກຳ)

ຮັບຈ້າງ:
7.1 =ຂົງເຂດກະສິກຳ
7.2 =ຂົງເຂດອື່ນໆ (ທີ່ບໍ່ແມ່ນກະສິກຳ)

8 = ອອກເງິນໃຫ້ຢືມ

ສຳລັບຜູ້ທີ່ບໍ່ມີລາຍຮັບ:
9 =ກຽມເຂົ້າໂຮງຮຽນ (0-6 ປີ)
10 =ຮັກຮຽນ
11 =ກຳລັງຊອກວຽກ
12 =ແມ່ບ້ານ
13 =ບຳນານ
14 =ບໍ່ໄດ້ເຮັດວຽກຍ້ອນຄວາມພິການ

ພາກທີ 2: ສະພາບທີ່ຢູ່ອາໄສຂອງຄົວເຮືອນ

ກະລຸນບອກ ກ່ຽວກັບສະພາບທີ່ຢູ່ອາໄສຂອງຄົວເຮືອນທ່ານ

	ຄຳຖາມ	ຄຳຕອບ			
			**ແອ້ມ**	**ຫຼັງຄາ**	**ພື້ນ**
1	ເຮືອນຂອງທ່ານແມ່ນສ້າງດ້ວຍ.........	1<br>2<br>3<br>4<br>5<br>6	= ເຟືອງ<br>= ດິນໜຽວ<br>= ດິນຈີ່<br>= ໄມ້<br>= ຊີມັງ<br>= ອື່ນໆ	= ເຟືອງ<br>= ສັງກະສີ<br>= ກະເບື້ອງ<br>= ໄມ້<br>= ອື່ນໆ	= ກະໂລ<br>= ຊີມັງ<br>= ດິນໜຽວ<br>= ໄມ້ປ່ອງ<br>= ໄມ້<br>= ອື່ນໆ
2	ເຮືອນທ່ານມີຫ້ອງນ້ຳບໍ່?	1<br>0	= ມີ<br>= ບໍ່ມີ		
3	ແຫຼ່ງນ້ຳດື່ມຕົ້ນຕໍ	1<br>2<br>3<br>4<br>5<br>6	= ຊືນໍາຕຸກ<br>= ນໍາກ໋ອກ<br>= ນໍາສ້າງ, ນໍາບາດານ<br>= ແມ່ນໍາ, ເຂືອນ, ຫ້ວຍ<br>= ນໍາຝົນໂຕ່ງໃສ່ອ່າງໄວ້<br>= ອືນໆ		
4	ແຫຼ່ງນ້ຳໃຊ້ຕົ້ນຕໍ	1<br>2<br>3<br>4<br>5<br>6	= ຊື້ນໍາຕຸກ<br>= ນໍາກ໋ອກ<br>= ນໍາສ້າງ, ນໍາບາດານ<br>= ແມ່ນໍາ, ເຂື້ອນ, ຫ້ວຍ<br>= ນໍາຝົນໂຕ່ງໃສ່ອ່າງໄວ້<br>= ອື້ນໆ		
5	ພະລັງງານຕົ້ນຕໍທີ່ໃຊ້ສຳລັບແສງສະຫວ່າງ ໃນຄົວເຮືອນຂອງທ່ານ	1<br>2<br>3<br>4<br>5<br>6	= ໄຟຟ້າ<br>= ແກັດ<br>= ນໍາມັນ<br>= ທຽນ<br>= ໄມ້<br>= ອື້ນໆ		
6	ພະລັງງານຕົ້ນຕໍທີ່ໃຊ້ສຳລັບການແຕ່ງກິນ ໃນຄົວເຮືອນຂອງທ່ານ	1<br>2<br>3<br>4<br>5	= ໄຟຟ້າ<br>= ແກັດ<br>= ນໍາມັນ<br>= ຟືນ, ຖ່ານ<br>= ອື້ນໆ		

ພາກທີ 3: ເນື້ອທີ່ການຜະລິດ, ຊັບສິນ ແລະ ສັດລ້ຽງເພື່ອການຜະລິດ, ເຄື່ອງໃຊ້ຍາວນານຂອງຄົວເຮືອນ

ກະລຸນາບອກ ກ່ຽວກັບຊັບສິນ ແລະ ສັດລ້ຽງເພື່ອການກະສິກຳ ແລະ ເຄື່ອງໃຊ້ຍາວນານ

	ຄຳຖາມ	ຄຳຕອບ	
1	ກະລຸນາບອກ ເນື້ອທີ່ການຜະລິດກະສິກຳ ທີ່ຄົວເຮືອນຂອງທ່ານມີ	ຕາແມັດ	1 = ດິນທີ່ເປັນເຈົ້າຂອງ (ຊື້ເອງ)<br>2 = ດິນທີ່ເປັນເຈົ້າຂອງ (ດິນມູນ)<br>3 = ດິນເຊົ່າຄົນອື່ນ<br>4 = ດິນໃຫ້ຄົນອື່ນເຊົ່າ<br>5 = ດິນປະເປັນເຮື້ອ<br>6 = ດິນອື່ນໆ
2	ກະລຸນາບບອກ ຊັບສິນທີ່ຄົວເຮືອນທ່ານມີ	ຈຳນວນ	1 = ລົດແທັກເຕີ (ຊື້ເອງ)<br>2 = ລົດແທັກເຕີ (ຊື້ຮ່ວມກັບຄົນອື່ນ)<br>3 = ລົດບັນທຸກ<br>4 = ລົດໄຖນາ<br>5 = ຈັກປັ່ນເຂົ້າ<br>6 = ໂຮງສີເຂົ້າ
3	ກະລຸນາບອກ ຈຳນວນສັດລ້ຽງທີ່ຄົວເຮືອນທ່ານມີ	ຈຳນວນ	1 = ຄວາຍ<br>2 = ງົວ<br>3 = ຫມູ<br>4 = ແບ້<br>5 = ສັດປີກ
4	ຄົວເຮືອນຂອງທ່ານມີ .... ບໍ່?	ຈຳນວນ	1 = ລົດຖີບ<br>2 = ລົດຈັກ<br>3 = ລົດໃຫຍ່ (ບໍ່ນັບລົດບັນທຸກ)
5	ຄົວເຮືອນຂອງທ່ານມີ .... ບໍ່? ທີ່ຍັງສາມາດໃຊ້ການໄດ້ໃນປະຈຸບັນ.	ຈຳນວນ	1 = ໂທລະທັດ<br>2 = ໂທລະສັບ<br>3 = ຕູ້ເຢັນ<br>4 = ຫມໍ້ໄຟຟ້າ<br>5 = ກາຕົ້ມນຳໄຟຟ້າ<br>6 = ຕູ້ຊັກຜ້າ<br>7 = ຕູ້ແຊ່<br>8 = ເຕົາແກັດ<br>9 = ພັດລົມ

ພາກທີ 4: ລາຍຮັບ

ກະລຸນາບອກ ກ່ຽວກັບລາຍຮັບທີ່ຄົວເຮືອນຂອງທ່ານມີ ໃນໄລຍະ 12 ເດືອນຜ່ານມາ

ແຫຼ່ງລາຍຮັບ	ຜົນຜະລິດ 12 ເດືອນຜ່ານມາ	ຂາຍ	ກິໂລລະ	ມູນຄ່າຂາຍທັງໝົດ	ຕົ້ນທຶນອື່ນໆ ເຊັ່ນ: ຝຸ່ຍ , ແຮງງານ
1 ເຂົ້າ	ກິໂລ	ກິໂລ	ກີບ	ກີບ	ກີບ

ແຫຼ່ງລາຍຮັບ	ມູນຄ່າຂາຍ 12 ເດືອນຜ່ານມາ	ຕົ້ນທຶນອື່ນໆ ເຊັ່ນ: ຝຸ່ຍ, ແຮງງານ, ຄ່າອາຫານສັດ
2 ຜັກ	ກີບ	ກີບ
3 ໝາກໄມ້	ກີບ	ກີບ
4 ສັດລ້ຽງ	ກີບ	ກີບ
5 ຫັດຖະກຳ	ກີບ	ກີບ
6 ຢາງພາລາ	ກີບ	ກີບ
7 ອ້ອຍ	ກີບ	ກີບ
8 ໄມ້ວິກ	ກີບ	ກີບ
9 ມັນຕົ້ນ	ກີບ	ກີບ
10 ອື່ນໆ	ກີບ	ກີບ

ແຫຼ່ງລາຍຮັບ	ມູນຄ່າຂາຍ 12 ເດືອນຜ່ານມາ
11 ຫາປາ	ກີບ
12 ເຄື່ອງປ່າຂອງດົງ	ກີບ
13 ເຜົາຖ່ານ	ກີບ
14 ອື່ນໆ	ກີບ

ແຫຼ່ງລາຍຮັບ	ລາຍໄດ້ 12 ເດືອນຜ່ານມາ
15 ເງິນຮັບຈ້າງ	ກີບ
16 ເງິນເດືອນ	ກີບ
17 ດອກເບ້ຍທະນາຄານ	ກີບ
18 ຄ້າຂາຍ	ກີບ
19 ເງິນໂອນມາ	ກີບ
20 ຄ່າເຊົ່າ	ກີບ
21 ເງິນບຳນານ	ກີບ
22 ອື່ນໆ………………	ກີບ

ພາກທີ 5: ລາຍຈ່າຍ

ກະລຸນາບອກ ກ່ຽວກັບລາຍຈ່າຍໃນຄົວເຮືອນຂອງທ່ານ ຕໍ່ເດືອນ ຫຼືຕໍ່ປີ (ຕອບຢ່າງດຽວ)

ແຫຼ່ງລາຍຈ່າຍ		ມູນຄ່າຈ່າຍ (ກີບ)	
		1 ເດືອນ	1 ປີ
1	ການເດີນທາງ, ການຂົນສົ່ງ (ຄ່ານໍ້າມັນ, ...)		
2	ການສຶກສາ		
3	ພະລັງງານ (ໄຟຟ້າ, ແກັດ, ຟືນ, ຖ່ານ,...)		
4	ນໍ້າ		
5	ໂທລະສັບ		
6	ເສື້ອຜ້າ, ເຮັດຜົມ, ເຄື່ອງສຳອາງ,...		
7	ຮັກສາສຸຂະພາບ		
8	ອາຫານ		
9	ໂອນເງິນໄປ		
10	ຄ່າເຊົ່າ		
11	ໃສ່ຊອງງານດອງ		
12	ບໍລິຈາກເຮັດບຸນ		
13	ຄ່າສະມາຄົມ, ເກັບສະຕິຕ່າງໆ...		
14	ຄ່າສ້ອມແປງຕ່າງໆ		
15	ລົງທຶນຄ້າຂາຍ (ຫຼືຊື້ສັດລ້ຽງ)		
16	ອື່ນໆ...................................		

ພາກທີ 6: ການກູ້ຢືມ

ກະລຸນາບອກ ກ່ຽວກັບການກູ້ຢືມເງິນຂອງຄົວເຮືອນທ່ານ ແຕ່ປີ 2012 ເປັນຕົ້ນມາ

	ແຫຼ່ງກູ້ຢືມ<br>1 = ທະນາຄານ<br>2 = ສະຖາບັນການເງິນ<br>3 = ການເງິນຈຸລະພາກ<br>4 = ສະມາຄົມຕ່າງໆ<br>5 = ເງິນກູ້ນອກລະບົບ<br>6 = ກອງທຶນບ້ານ<br>7 = ເພື່ອນ, ພີ່ນ້ອງ<br>8 = ອື່ນໆ	ເດືອນ, ປີ ທີ່ຢືມ	ມູນຄ່າຢືມ (ກີບ)	ຈຸດປະສົງທີ່ຢືມ<br>1 = ເຮັດກະສິກຳ<br>2 = ລ້ຽງສັດ<br>3 = ຮັກສາສຸຂະພາບ<br>4 = ການສຶກສາ<br>5 = ໃຊ້ຈ່າຍ<br>6 = ລົງທຶນຄ້າຂາຍ<br>7 = ອື່ນໆ	ດອກເບ້ຍຕໍ່ປີ (%)	ເດືອນ, ປີ ກຳນົດສົ່ງ	ມູນຄ່າທີ່ຍັງ ເປັນໜີ້ (ກີບ)
1	2	3	4	5	6	7	8
1							
2							
3							

ພາກທີ 7: ການຝາກເງິນ

ກະລຸນາບອກ ກ່ຽວກັບການຝາກເງິນຂອງຄົວເຮືອນທ່ານ ແຕ່ປີ 2012 ເປັນຕົ້ນມາ

	ແຫຼ່ງຝາກເງິນ<br>1 = ທະນາຄານ<br>2 = ສະຖາບັນການເງິນ<br>3 = ການເງິນຈຸລະພາກ<br>4 = ສະມາຄົມຕ່າງໆ<br>5 = ເງິນກູ້ນອກລະບົບ<br>6 = ເພື່ອນ, ພີ່ນ້ອງ<br>7 = ອື່ນໆ	ເດືອນ, ປີ ທີ່ຝາກ	ມູນຄ່າຝາກ	ດອກເບ້ຍຕໍ່ປີ (%)
1	2	3	4	5
1				
2				
3				

ພາກທີ 8: ການອອມເປັນວັດຖຸ

ກະລຸນາບອກ ກ່ຽວກັບການອອມເງິນ ໃນຮູບແບບວັດຖຸ

	ລາຍການ	ມູນຄ່າ (ກີບ)
1	2	3
1	ຄຳ	
2	ເພັດ	

ພາກທີ 9: ລະບົບປະກັນສຸຂະພາບຊຸມຊົນ (ກປສຊ)

ກະລຸນາບອກ ກ່ຽວກັບ ການປະກັນ ກປສຊ ຂອງຄົວເຮືອນທ່ານ

	ຄຳຖາມ	ຄຳຕອບ
1	ຄົວເຮືອນທ່ານໄດ້ເປັນສະມາຊິກຂອງ ກປສຊ ບໍ່?	1 = ເປັນສະມາຊິກ (ຂ້າມໄປ ຂໍ້4)<br>2 = ເຄີຍເປັນສະມາຊິກ (ຂ້າມໄປ ຂໍ້3)<br>3 = ບໍ່ແມ່ນສະມາຊິກ
2	ທ່ານເຄີຍໄດ້ຍິນກ່ຽວກັບ ລະບົບ ກປສຊ ບໍ່?	1 = ເຄີຍ<br>0 = ບໍ່ (ຂ້າມໄປ ພາກທີ 10)
3	ເປັນຫຍັງທ່ານຈຶ່ງບໍ່ເຂົ້າລະບົບປະກັນສຸຂະພາບ ກປສຊ?	ລຽງລຳດັບ 1-3<br>1 = ຂໍ້ມູນບໍ່ພຽງພໍ<br>2 = ຄ່າປະກັນແພງໂພດ<br>3 = ບໍ່ມີເງິນ<br>4 = ການໃຫ້ບໍລິການຂອງໂຮງໝໍຮ່ວມສັນຍາບໍ່ດີ<br>5 = ການບໍລິຫານຂອງລະບົບ ກປສຊ ບໍ່ໜ້າເຊື່ອຖື<br>6 = ປະໂຫຍດທີ່ຈະໄດ້ຮັບຈາກການປະກັນບໍ່ນ່າເຊື່ອຖື<br>7 = ອື່ນໆ…………………………………………
4	ທ່ານຮູ້ / ເຄີຍໄດ້ຮູ້ ກ່ຽວກັບ ລະບົບ ກປສຊ ໄດ້ແນວໃດ?	ລຽງລຳດັບ 1-3<br>1 = ຈາກສະມາຊິກຄົວເຮືອນ<br>2 = ຈາກໝູ່ເພື່ອນ<br>3 = ຈາກສະຖານຮັກສາຂະພາບ<br>4 = ຈາກພະນັກງານຂອງ ກປສຊ<br>5 = ຈາກສະມາຄົມອື່ນໆ<br>6 = ຈາກຫອງການບານ<br>7 = ຈາກສື່ຕາງໆ ເຊັ່ນ: ໂທລະທັດ, ວິທະຍຸ,…<br>8 = ຈາກສະມາຊິກ ກປສຊ ຄົນອື່ນໆ<br>9 = ອື່ນໆ………………………………………
5	ທ່ານເຄີຍເຊົາເປັນສະມາຊິກ ແລ້ວກັບມາເປັນໃຫມ່ອີກບໍ່?	1 = ເຄີຍ<br>0 = ບໍ່ (ຂ້າມໄປ ຂໍ້7)
6	ຖ້າເຄີຍ, ກະລຸນາບອກ ເດືອນ/ປີ ແຕ່ລະໄລຍະຂອງການເປັນສະມາຊິກ ກປສຊ	ແຕ່ ………………ເຖິງ……………….<br>ແຕ່ ………………ເຖິງ……………….
7	ທ່ານເລີ່ມເປັນສະມາຊິກແຕ່ ເດືອນ/ປີ ໃດ?	
8	ທ່ານເຊົາເປັນສະມາຊິກແຕ່ ເດືອນ/ປີ ໃດ?	
9	ທ່ານຈ່າຍ / ໄດ້ຈ່າຍຄ່າ ປກສຊ ທຸກເດືອນປົກກະຕິບໍ່?	1 = ປົກກະຕິ (ຂ້າມໄປ ຂໍ້11)<br>0 = ບໍ່ປົກກະຕິ
10	ຖ້າ ຈ່າຍບໍ່ເປັນປົກກະຕິ, ຍ້ອນຫຍັງ?<br><br>***<u>ສໍາລັບຜູ້ເຄີຍເປັນສະມາຊິກ:</u>*** ເປັນຫຍັງທ່ານຈຶ່ງເຊົາເປັນສະມາຊິກ?	ລຽງລຳດັບ 1-3<br>1 = ບໍ່ມີຜູ້ມາເກັບເງິນ<br>2 = ຄ່າປັນກັນແຕ່ລະເດືອນແພງໂພດ<br>3 = ບໍ່ມີເງິນ<br>4 = ການໃຫ້ບໍລິການຂອງໂຮງໝໍຮ່ວມສັນຍາບໍ່ດີ<br>5 = ການບໍລິຫານຂອງລະບົບ ກປສຊ ບໍ່ໜ້າເຊື່ອຖື<br>6 = ປະໂຫຍດທີ່ຈະໄດ້ຮັບຈາກການປະກັນບໍ່ນ່າເຊື່ອຖື<br>7 = ອື່ນໆ…………………………………………

	ຄຳຖາມ	ຄຳຕອບ
11	ທ່ານຮູ້ຈັກຄະນະບໍລິຫານຂອງກອງທຶນ ກປສຊ ຂັ້ນບ້ານ ແລະ ເມືອງຂອງທ່ານບໍ່?	ຮູ້ ບໍ່<br>1 0 ຫົວໜ້າ ກປສຊ ຂັ້ນບ້ານ/ເມືອງ<br>1 0 ພະນັກງານ ກປສຊ ຂັ້ນບ້ານ/ເມືອງ
12	ທ່ານຮູ້ຈັກສະມາຊິກ ກປສຊ ໃນຂອບເຂດບ້ານຂອງທ່ານຈັກຄົນ?	……………………..…. ຄົນ
13	ທ່ານຄິດວ່າ ກອງທຶນ ກປສຊ ໃຫ້ປະໂຫຍດຫຍັງແດ່ແກ່ບັນດາສະມາຊິກ ກໍ່ຄືຄົວເຮືອນຂອງທ່ານ?	ລຽງລຳດັບ 1-3<br>☐ 1= ທຸກຄັ້ງທີ່ບໍ່ສະບາຍ ແມ່ນສາມາດໃຊ້ບັດ ປກສຊ ເພື່ອປິ່ນ<br>☐ 2= ຊ່ວຍຫຼຸດຜ່ອນຄ່າໃຊ້ຈ່າຍປິ່ນປົວສຸຂະພາບ<br>☐ 3= ຈຳເປັນ ໂດຍສະເພາະໃນກໍລະນີ ທີ່ຕ້ອງໄດ້ປິ່ນປົວສຸຂະ<br>☐ 4= ຊ່ວຍໃຫ້ສຸຂະພາບໂດຍລວມດີຂຶ້ນກວ່າເກົ່າ<br>☐ 5= ອື່ນໆ…………...……………………….

ພາກທີ 10: ສຸຂະພາບ ແລະ ການເຂົ້າເຖິງສະຖານທີ່ຮັກສາສຸຂະພາບ

ກະລຸນາບອກ ກ່ຽວກັບສຸຂະພາບຂອງທ່ານ ແລະ ສະມາຊິກໃນຄົວເຮືອນ

	ຄຳຖາມ	ຄຳຕອບ
1	ສະຖານທີ່ຮັກສາສຸຂະພາບທີ່ໃກ້ເຮືອນທ່ານທີ່ສຸດ ແມ່ນຢູ່ໄກປານໃດ?	………………… ກິໂລແມັດ
2	ສ່ວນໃຫຍ່ແລ້ວ ທ່ານ/ສະມາຊິກຄົວເຮືອນ ເດີນທາງໄປຮັກສາສຸຂະພາບ ດ້ວຍວິທີໃດ?	1 = ຍ່າງ<br>2 = ລົດຈັກ<br>3 = ລົດໃຫຍ່<br>4 = ເຊົ່າ/ຢືມ ລົດຈາກຄົນອື່ນ<br>5 = ອື່ນໆ……………...………………
3	ໃນໄລຍະ 2 ປີມານີ້ ມີສະມາຊິກຄົວເຮືອນຄົນໃດນອນໂຮງໝໍບໍ່?	ບໍ່ ມີ<br>0 1 ຖ້າມີ, ……………….ຄົນ
4	ໃນໄລຍະ 2 ປີມານີ້ ມີສະມາຊິກຄົວເຮືອນຄົນໃດ ທີ່ທ່ານຄິດວ່າ ຄວນຈະນອນປິ່ນປົວຢູ່ສະຖານທີ່ຮັກສາສຸຂະພາບ, ແຕ່ບໍ່ໄດ້ຮັບການປິ່ນປົວ ຍ້ອນບໍ່ມີເງິນບໍ່?	ບໍ່ ມີ<br>0 1 ຖ້າມີ, ……………….ຄົນ

5: ກະລຸນາລະບຸລະອຽດ ສະມາຊິກຜູ້ທີ່ໄດ້ຮັບການປິ່ນປົວສຸຂະພາບ ໃນໄລຍະ 12 ເດືອນຜ່ານມາ

ສໍາລັບສະມາຊິກ ແລະ ເຄີຍເປັນສະມາຊິກ ຫຼັງເດືອນ 9/2015

ລະຫັດບຸກຄົນ	ປະເພດການປິ່ນປົວ<br><br>1=ນອນໂຮງໝໍ<br>2=ປິ່ນປົວເຂດນອກ<br>3=ປິ່ນປົວພື້ນເມືອງ	ຄ່າການບໍລິການປິ່ນປົວ<br><br>(ກີບ)<br>*ເຊັ່ນ: ຄ່າຕຽງນອນໂຮງໝໍ, ຄ່າກວດ, ຄ່າເຮັດປີ້, ,...*	ຄ່າຢາ<br><br>(ກີບ)	ຄ່າລາຍຈ່າຍ ອື່ນໆ<br><br>(ກີບ)<br>*ເຊັ່ນ: ຄ່າເດີນທາງ , ຄ່າອາຫານ, ຄ່າໃຊ້ຈ່າຍໃນການມານອນເຝົ້າ,...*	ທ່ານເອົາເງິນມາແຕ່ໃສ?<br><br>1=ລາຍຮັບປະຈໍາ<br>2=ເງິນທ້ອນໄວ້<br>3=ຢືມ<br>4=ຂາຍສິ່ງທີ່ມີ<br>5=ອື່ນໆ	ກອງທຶນ ກປສຊ ຈ່າຍຄ່າປິ່ນປົວໃຫ້ທ່ານບໍ່?<br><br>*ຄ່າບໍລິການ + ຄ່າຢາ*<br>0=ບໍ່<br>1=ຈ່າຍບາງສ່ວນ<br>2=ຈ່າຍໃຫ້ໝົດ	ໃຫ້ທ່ານປະເມີນການໃຫ້ບໍລິການຂອງລະບົບ ກປສຊ<br><br>1=ພໍໃຈຫຼາຍ<br>2=ພໍໃຈ<br>3=ປານກາງ<br>4=ບໍ່ພໍໃຈ<br>5=ບໍ່ພໍໃຈຫຼາຍ
1							
2							
3							
4							
5							
6							
7							
8							
9							
10							

ພາກທີ 11: ເຄືອຂ່າຍທາງສັງຄົມ

ກະລຸນາບອກ ກ່ຽວກັບເຄືອຂ່າຍທາງສັງຄົມຂອງທ່ານ ແລະ ສະມາຊິກໃນຄົວເຮືອນຂອງທ່ານ

	ຄຳຖາມ	ຄຳຕອບ
1	ຫົວໜ້າຄົວເຮືອນທ່ານອາໄສຢູ່ບ້ານນີ້ດົນປານໃດ?	................................ປີ
2	ນອກຈາກກອງທຶນ ກປສຊ, ທ່ານ ແລະ ສະມາຊິກຄົວເຮືອນຄົນອື່ນໆ ຍັງເປັນສະມາຊິກຂອງສະມາຄົມ, ກອງທຶນ, ຫຼືອົງການຈັດຕັ້ງອື່ນໆອີກບໍ່?	1 _ ຄະນະພັກບ້ານ<br>2 _ ອຳນາດການປົກຄອງບ້ານ<br>3 _ ສະຫະພັນແມ່ຍິງບ້ານ<br>4 _ ຊາວໜຸ່ມບ້ານ<br>5 _ ກຳມະບານບ້ານ<br>6 _ ແນວໂຮມບ້ານ<br>7 _ ປກສ ບ້ານ<br>8 _ ກອງຫຼອນບ້ານ<br>9 _ ສະມາຄົມພໍ່ແມ່ນັກຮຽນ<br>10 _ ກຸ່ມລ້ຽງສັດ<br>11 _ ກຸ່ມປູກເຂົ້າ<br>12 _ ກອງທຶນບ້ານ<br>13 _ ການເງິນຈຸລະພາກ<br>14 _ ກຸ່ມກິລາ<br>15 _ ອົງການສາກົນ<br>16 _ ອື່ນໆ..............................
3	ຖ້າວ່າ ມີໂຄງການຈະຖືກຈັດຕັ້ງປະຕິບັດໃນບ້ານນີ້ ເຊິ່ງເປັນໂຄງການທີ່ບໍ່ໄດ້ໃຫ້ຜົນປະໂຫຍດຕໍ່ທ່ານ ແລະ ສະມາຊິກຄົວເຮືອນທ່ານໂດຍກົງ ແຕ່ມີປະໂຫຍດຕໍ່ຄົນໃນຂອບເຂດບ້ານຂອງທ່ານໂດຍລວມ, ຖາມວ່າ ທ່ານຍິນດີທີ່ຈະປະກອບສ່ວນທາງດ້ານ **"ເວລາ"** ຫຼື **"ເງິນ"** ເຂົ້າໃນໂຄງການນີ້ບໍ່?	ໃຫ້ ບໍ່<br>1 0 **"ເວລາ"**<br>1 0 **"ເງິນ"**
4	ໂດຍສ່ວນໃຫຍ່ແລ້ວ ທ່ານ ແລະ ສະມາຊິກຄົວເຮືອນຄົນອື່ນໆ ໄດ້ຮັບຮູ້ຂໍ້ມູນຂ່າວສານກ່ຽວກັບ ການເມືອງ, ໂຄງການຂອງລັດຖະບານ, ໂຄງການສາກົນຕ່າງໆ, ສະມາຄົມຕ່າງໆ, ແລະ ຂໍ້ມູນກ່ຽວກັບບ້ານ ຫຼື ຊຸມຊົນຂອງທ່ານເອງ ໄດ້ແນວໃດ?	ລຽງລຳດັບ 1 - 3<br>☐ 1 = ພີ່ນ້ອງ, ໝູ່ເພື່ອນ<br>☐ 2 = ກະດານຂ່າວຫ້ອງການບ້ານ<br>☐ 3 = ຕະຫຼາດ<br>☐ 4 = ວິທະຍຸ<br>☐ 5 = ໂທລະທັດ<br>☐ 6 = ສະພາກາເຟ/ສະມາຄົມຕ່າງໆ<br>☐ 7 = ນາຍບ້ານ<br>☐ 8 = ຫ້ອງການຂອງລັດ<br>☐ 9 = ອົງການສາກົນ (NGOs)<br>☐ 10 = ອື່ນໆ.........................
5	ໃນໄລຍະ 12 ເດືອນຜ່ານມາ ຄົວເຮືອນຂອງທ່ານເຄີຍເອົາເງິນໃຫ້ຄົນອື່ນຢືມບໍ່? (ຖ້າເຄີຍ, ຈັກຄັ້ງ?)	1 = ເຄີຍ (............ຄັ້ງ)<br>0 _ ບໍ່
6	ໂດຍສະເລ່ຍແລ້ວ, ຄົວເຮືອນຂອງທ່ານໃຫ້ຢືມເທົ່າໃດຕໍ່ຄັ້ງ?	................................ກີບ
7	ເຮືອນຂອງທ່ານຢູ່ໄກຈາກຕະຫຼາດປານໃດ?	................................ກິໂລແມັດ

ພາກທີ 12: ເຫດການສຸກເສີນຕ່າງໆ

ກະລຸນບອກ ກ່ຽວກັບເຫດການສຸກເສີນກ່ຽວກັບທ່ານ ແລະ ສະມາຊິກຄົວເຮືອນຂອງທ່ານ ແຕ່ປີ 2012 ເປັນຕົ້ນມາ

ເຫດການສຸກເສີນ	ໄລຍະເວລາຂອງເຫດການ				ຄວາມເສຍຫາຍຄິດໄລ່ເປັນເງິນເທົ່າໃດ? (ກີບ)
	ເລີ່ມແຕ່		ຈົນເຖິງ		
	ເດືອນ	ປີ	ເດືອນ	ປີ	
**1. ເສຍຊີວິດ**<br>a. ເປັນໃຜ?:...<br>b. ອາຍຸ: ....<br>c. ອາຊີບ:..					ຄ່າປິ່ນປົວ + ງານສົບ
**2. ອຸປະຕິເຫດ**<br>a. ເປັນໃຜ?:...<br>b. ອຸປະຕິເຫດຫຍັງ?:					ຄ່າປິ່ນປົວ + ສ້ອມແປງ
**3. ພະຍາດໂລກລະບາດໃນສັດ**<br>1. ປະເພດສັດ:...<br>ຈຳນວນທີ່ເສຍຫາຍ: ....<br>2. ປະເພດສັດ:...<br>ຈຳນວນທີ່ເສຍຫາຍ: ....<br>3. ປະເພດສັດ:...<br>ຈຳນວນທີ່ເສຍຫາຍ:					ຄ່າປິ່ນປົວ + ສັດຕາຍ
**4. ຄວາມເສຍຫາຍໃນຜົນລະປູກ ຍ້ອນໄພທຳມະຊາດ ແລະ ສັດຕູພືດ**<br>1. ປະເພດເຄື່ອງປູກ:......................................<br>ຈຳນວນທີ່ເສຍຫາຍ: ....<br>2. ປະເພດເຄື່ອງປູກ:......................................<br>ຈຳນວນທີ່ເສຍຫາຍ: ....<br>3. ປະເພດເຄື່ອງປູກ:......................................<br>ຈຳນວນທີ່ເສຍຫາຍ:					ຄວາມເສຍຫາຍເປັນເງິນ
**5. ໄພພິບັດອື່ນໆ**<br>a. ໄພພິບັດຫຍັງ?:...<br>b. ເຫດການເກີດຂຶ້ນແກ່ຍາວດົນປານໃດ:					ຄ່າເສຍຫາຍ ເຊັ່ນ: ສ້ອມແປງເຮືອນ,....
**6. ຖືກລັກ**<br>a. ປະເພດທີ່ຖືກລັກ:..					
**7. ຈັດງານດອງ:**....................................ຄັ້ງ					
**8. ຂາຍ:** ດິນ, ເຮືອນ, ລົດຈັກ, ລົດໃຫຍ່					
**9. ຊື້:** ດິນ, ເຮືອນ, ລົດຈັກ, ລົດໃຫຍ່, ລົດໄຖນາ, ຈັກປັ່ນເຂົ້າ ...					
**10. ອື່ນໆ**					

ພາກທີ 13: ການທົດລອງ Conjoint Experiment

(ກ່ອນທີ່ຈະເລີ່ມເຮັດການທົດລອງ ທີມງານຕ້ອງໄດ້ອ່ານຂໍ້ຄວາມດັ່ງລຸ່ມນີ້ໃຫ້ຜູ້ເຂົ້າຮ່ວມເຮັດການທົດລອງແຕ່ລະຄົນຟັງ. ໃນຕອນທ້າຍ ທີມງານຕ້ອງໄດ້ກວດກາຄືນວ່າ ຜູ້ເຂົ້າຮ່ວມເຂົ້າໃຈກ່ຽວກັບຂັ້ນຕອນຕ່າງໆຂອງການທົດລອງແລ້ວ).

ທີມເຮັດການຄົ້ນຄວ້າພວກເຮົາ ມີຈຸດປະສົງເພື່ອຢາກສົ່ງເສີມໃຫ້ປະຊາຊົນທີ່ບໍ່ມີອາຊີບແນ່ນອນ ມີສຸຂະພາບທີ່ດີຂຶ້ນ ໂດຍຜ່ານທາງ ການເຂົ້າຮ່ວມລະບົບປະກັນສຸຂະພາບ ກປສຊ ເຊິ່ງເປັນກອງທຶນທີ່ຈັດຕັ້ງປະຕິບັດຢູ່ຂັ້ນເມືອງ.

ຂ້າງລຸ່ມນີ້ ແມ່ນເຫດການສົມມຸດ ສຳລັບກອງທຶນປະກັນສຸຂະພາບຈຳລອງຂອງ ກປສຊ. ຫລັງຈາກທີ່ທ່ານຮັບຊາບ ແລະ ເຂົ້າໃຈກ່ຽວກັບເຫດການສົມມຸດແລ້ວ ຂໍໃຫ້ທ່ານ ກະລຸນາໃຫ້ລຳດັບຄວາມເພິ່ງພໍໃຈ ໃສ່ 3 ທາງເລືອກໃນແຕ່ລະຊຸດທາງເລືອກ (choice task).

"ທີມຄົ້ນຄວ້າພວກເຮົາ ຕ້ອງການທີ່ຈະປັບປຸງລະບົບປະກັນສຸຂະພາບ ກປສຊ ດ້ວຍນະໂຍບາຍຕ່າງໆທີ່ຫລາກຫລາຍ. ສົມມຸດວ່າ ລະບົບປະກັນສຸຂະພາບຈຳລອງ ຂອງ ກປສຊ ຄຸ້ມຄອງທັງການບໍລິການຄົນໄຂ້ເຂດໃນ ແລະ ເຂດນອກ, ເຊິ່ງຜູ້ທີ່ໃຊ້ບໍລິການຈຳເປັນຕ້ອງໄດ້ຮັບການປິ່ນປົວໃນໂຮງໝໍທີ່ໄດ້ເຮັດສັນຍາກັບທາງລະບົບເທົ່ານັ້ນ ເຊິ່ງໄດ້ແກ່ ສຸກສາລາ ແລະ ໂຮງໝໍເມືອງຂອງທ່ານ. ທ່ານທີ່ເຂົ້າຮັບການບໍລິການປິ່ນປົວ ສາມາດໄປປິ່ນປົວຕໍ່ທີ່ໂຮງໝ ໍແຂວງ ແລະ ໂຮງໝໍພາກໄດ້ ຖ້າຫາກວ່າມີໜັງສືນຳສົ່ງຈາກໂຮງໝໍເມືອງຂອງທ່ານ. ສ່ວນເງິນປະກັນ ທ່ານສາມາດຈ່າຍເປັນເດືອນ ຫລື ເປັນປີໄດ້. ສຳລັບ ຜູ້ເຂົ້າໃຫມ່ ແມ່ນສາມາດເລີ່ມຮັບການບໍລິການຈາກສະຖານບໍລິການສຸຂະພາບຄູ່ສັນຍາໄດ້ ພາຍຫລັງຈາກການເສຍເງິນປະກັນເປັນເວລາ 3 ເດືອນຕໍ່ເນື່ອງ. ສົມມຸດວ່າ ຖ້າຫາກທຸກເມືອງມີສະມາຊິກກອງທຶນຮອດ 500 ຫລື ຫລາຍກວ່ານັ້ນ ໂຮງໝໍເມືອງ ໃນນາມຜູ້ຈັດຕັ້ງປະຕິບັດຂັ້ນເມືອງ ຈະສາມາດສ້າງກຳໄລຈາກການຈັດຕັ້ງປະຕິບັດລະບົບດັ່ງກ່າວ ເຮັດໃຫ້ຄຸນນະພາບການບໍລິການຄ່ອຍໆດີຂຶ້ນໃນໄລຍະຍາວ."

ສຳລັບການທົດລອງ, ຜູ້ເຂົ້າຮ່ວມເຮັດການທົດລອງແຕ່ລະທ່ານ ຈະໄດ້ຮັບຊຸດທາງເລືອກ (choice task) ທັງໝົດ 5 ຊຸດ. ເຊິ່ງໃນແຕ່ລະຊຸດ ປະກອບມີ 3 ທາງເລືອກ: 2 ທາງເລືອກຈຳລອງ (A ແລະ B) ແລະ 1 ທາງເລືອກເດີມ ທີ່ເປັນສະຖານະການຕົວຈິງຂອງກອງທຶນປະກັນສຸຂະພາບ ກປສຊ (Status quo). ເຊິ່ງໃນແຕ່ລະທາງເລືອກແມ່ນປະກອບດ້ວຍ 7 ຄຸນລັກສະນະຄືດັ່ງນີ້: ອັດຕາເງິນສົມທົບ, ກອງທຶນຄຸ້ມຄອງຄ່ານອນໂຮງໝໍ, ກອງທຶນຄຸ້ມຄອງຄ່າກວດດ້ານເຕັກນິກຕ່າງໆ ແລະ ບົ່ງມະຕິພະຍາດ, ກອງທຶນຄຸ້ມຄອງຄ່າຢາ, ກອງທຶນຄຸ້ມຄອງຄ່າປິ່ນປົວຍ້ອນອຸບັດຕິເຫດຈາກການສັນຈອນ, ກອງທຶນຄຸ້ມຄອງຄ່າເດີນທາງໄປໂຮງໝໍຕ່າງເມືອງຂອງຄົນເຈັບ, ແລະ ສ່ວນຫຼຸດສຳລັບການຈ່າຍເງິນສົມທົບໝົດປີລ່ວງໜ້າ.

ຮູບພາບສະແດງລະດັບຂອງແຕ່ລະຄຸນລັກສະນະ.

1.1	1 = 12,000<br>2-4 = 20,000<br>5-7 = 25,000<br>≥8 = 28,000<br>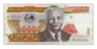<br>- 2,000	ອັດຕາເງິນສົມທົບ/ຄອບຄົວ/ເດືອນ ແມ່ນຫຼຸດຈາກອັດຕາໃນປະຈຸບັນ 2,000ກີບ, ຕົວຢ່າງ: 12,000ກີບ → 10,000ກີບ.
1.2	1 = 12,000<br>2-4 = 20,000<br>5-7 = 25,000<br>≥8 = 28,000<br>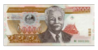	ອັດຕາເງິນສົມທົບ/ຄອບຄົວ/ເດືອນ ໃນປະຈຸບັນ.
1.3	1 = 12,000<br>2-4 = 20,000<br>5-7 = 25,000<br>≥8 = 28,000<br>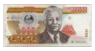<br>+ 2,000	ອັດຕາເງິນສົມທົບ/ຄອບຄົວ/ເດືອນ ແມ່ນເພີ່ມຈາກອັດຕາໃນປະຈຸບັນ 2,000ກີບ, ຕົວຢ່າງ: 12,000ກີບ → 14,000ກີບ.
1.4	1 = 12,000<br>2-4 = 20,000<br>5-7 = 25,000<br>≥8 = 28,000<br>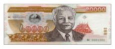<br>+ 4,000	ອັດຕາເງິນສົມທົບ/ຄອບຄົວ/ເດືອນ ແມ່ນເພີ່ມຈາກອັດຕາໃນປະຈຸບັນ 4,000ກີບ, ຕົວຢ່າງ: 12,000ກີບ → 16,000ກີບ.
2.2	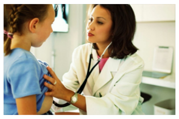	ຄຸ້ມຄອງ ຄ່າກວດດ້ານເຕັກນິກຕ່າງໆ, ຄ່າບົ່ງມະຕິພະຍາດ.
3.2	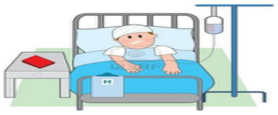	ຄຸ້ມຄອງ ຄ່າຫ້ອງນອນ (ທີ່ກອງທຶນກຳນົດໃຫ້).
4.2	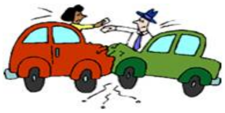	ຄຸ້ມຄອງ ຄ່າປິ່ນປົວຍ້ອນອຸປະຕິເຫດຈາກການສັນຈອນ.
5.1	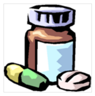	ຄຸ້ມຄອງ ຄ່າຢາທີ່ນອນໃນບັນຊີຢາພື້ນຖານທີ່ກຳນົດໂດຍກະຊວງສາທາ ສຳລັບແຕ່ລະລະດັບໂຮງໝໍແລ້ວ.
5.2	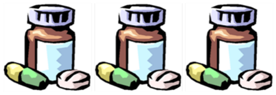	ຄຸ້ມຄອງ ຄ່າຢາທຸກຊະນິດ ທີ່ຈຳເປັນຕ້ອງໃຊ້ເພື່ອປິ່ນປົວ.
6.2	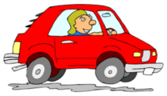	ຄຸ້ມຄອງ ຄ່າເດີນທາງໄປໂຮງໝໍຕ່າງເມືອງຂອງຄົນເຈັບ 1 ທ່ຽວ.
6.3	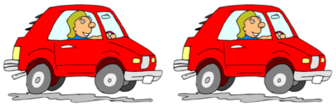	ຄຸ້ມຄອງ ຄ່າເດີນທາງໄປໂຮງໝໍຕ່າງເມືອງຂອງຄົນເຈັບ ໄປ-ກັບ.
7.2	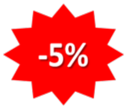	ຖ້າວ່າ ຈ່າຍເງິນສົມທົບໝົດປີ ຈະໄດ້ສ່ວນຫຼຸດ 5%.
7.3	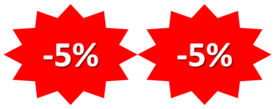	ຖ້າວ່າ ຈ່າຍເງິນສົມທົບໝົດປີ ຈະໄດ້ສ່ວນຫຼຸດ 10%.

ກະລຸນາໃຫ້ລຳດັບທາງເລືອກດັ່ງລຸ່ມນີ້ ຕາມຄວາມເພິ່ງພໍໃຈຂອງທ່ານ.

1= ພໍໃຈທີ່ສຸດ
2= ພໍໃຈປານກາງ
3= ພໍໃຈໜ້ອຍທີ່ສຸດ

<u>ຕົວຢ່າງ:</u>

	ທາງເລືອກ A	ທາງເລືອກ B	ທາງເລືອກເດີມ
ອັດຕາເງິນສົມທົບ	1 = 12,000<br>2-4 = 20,000<br>5-7 = 25,000<br>≥8 = 28,000<br>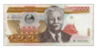<br>- 2,000	1 = 12,000<br>2-4 = 20,000<br>5-7 = 25,000<br>≥8 = 28,000<br>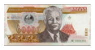<br>+ 2,000	1 = 12,000<br>2-4 = 20,000<br>5-7 = 25,000<br>≥8 = 28,000<br>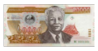
ສ່ວນຫຼຸດສຳລັບການຈ່າຍເງິນສົມທົບໝົດປີລ່ວງໜ້າ		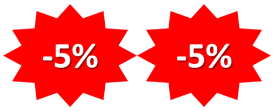	
ຄຸ້ມຄອງຄ່ານອນໂຮງໝໍ	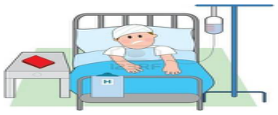		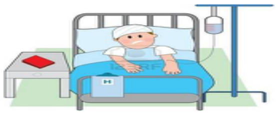
ຄຸ້ມຄອງຄ່າກວດດ້ານເຕັກນິກຕ່າງໆ ແລະ ບົ່ງມະຕິພະຍາດ		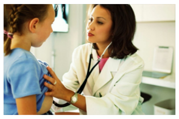	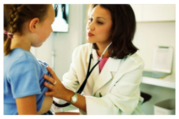
ກອງທຶນຄຸ້ມຄອງຄ່າຢາ	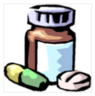	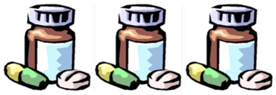	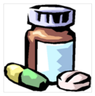
ຄຸ້ມຄອງຄ່າເດີນທາງໄປໂຮງໝໍຕ່າງເມືອງຂອງຄົນເຈັບ	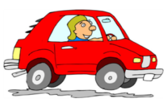	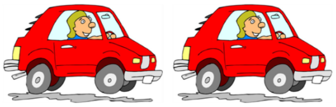	
ຄຸ້ມຄອງຄ່າປິ່ນປົວຍ້ອນອຸບັດຕິເຫດຈາກການສັນຈອນ	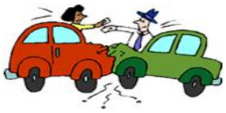	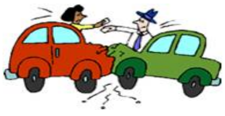	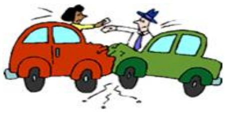
	1	3	2

ກະລຸນາໃຫ້ລຳດັບທາງເລືອກດັ່ງລຸ່ມນີ້ ຕາມຄວາມເພິ່ງພໍໃຈຂອງທ່ານ.

Choice set 75

ຊຸດທາງເລືອກ 1:

	ທາງເລືອກ A	ທາງເລືອກ B	ທາງເລືອກເດີມ
ຄຸ້ມຄອງຄ່າກວດດ້ານເຕັກນິກຕ່າງໆ ແລະ ບົ່ງມະຕິພະຍາດ		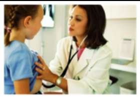	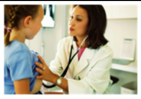
ຄຸ້ມຄອງຄ່າປິ່ນປົວຍ້ອນອຸບັດຕິເຫດ ຈາກການສັນຈອນ		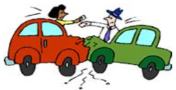	
ຄຸ້ມຄອງຄ່າເດີນທາງໄປໂຮງໝໍຕ່າງ ເມືອງຂອງຄົນເຈັບ		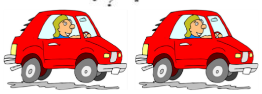	
ກອງທຶນຄຸ້ມຄອງຄ່າຢາ	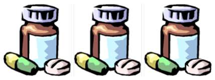	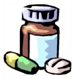	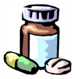
ອັດຕາເງິນສົມທົບ	1 = 12,000<br>2-4 = 20,000<br>5-7 = 25,000<br>≥8 = 28,000 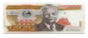 + 4,000	1 = 12,000<br>2-4 = 20,000<br>5-7 = 25,000<br>≥8 = 28,000 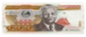 + 4,000	1 = 12,000<br>2-4 = 20,000<br>5-7 = 25,000<br>≥8 = 28,000 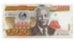
ຄຸ້ມຄອງຄ່ານອນໂຮງໝໍ	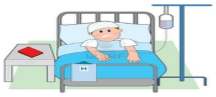	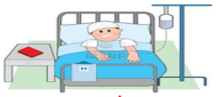	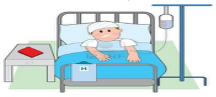
ສ່ວນຫຼຸດສຳລັບການຈ່າຍເງິນສົມທົບໝົດປີລ່ວງໜ້າ		-5%	
	☐	☐	☐

ຊຸດທາງເລືອກ 2:

	ທາງເລືອກ A	ທາງເລືອກ B	ທາງເລືອກເດີມ
ຄຸ້ມຄອງຄ່າກວດດ້ານເຕັກນິກຕ່າງໆ ແລະ ບົ່ງມະຕິພະຍາດ		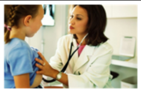	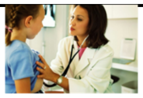
ຄຸ້ມຄອງຄ່າປິ່ນປົວຍ້ອນອຸບັດຕິເຫດ ຈາກການສັນຈອນ		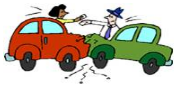	
ຄຸ້ມຄອງຄ່າເດີນທາງໄປໂຮງໝໍຕ່າງ ເມືອງຂອງຄົນເຈັບ		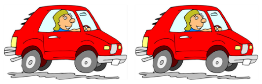	
ກອງທຶນຄຸ້ມຄອງຄ່າຢາ	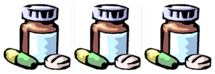	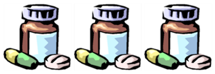	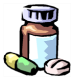
ອັດຕາເງິນສົມທົບ	1 = 12,000<br>2-4 = 20,000<br>5-7 = 25,000<br>≥8 = 28,000 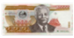	1 = 12,000<br>2-4 = 20,000<br>5-7 = 25,000<br>≥8 = 28,000 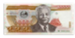 - 2,000	1 = 12,000<br>2-4 = 20,000<br>5-7 = 25,000<br>≥8 = 28,000 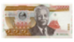
ຄຸ້ມຄອງຄ່ານອນໂຮງໝໍ			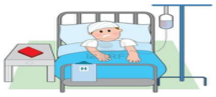
ສ່ວນຫຼຸດສຳລັບການຈ່າຍເງິນສົມທົບໝົດປີລ່ວງໜ້າ	-5%	-5%	
	☐	☐	☐

ຊຸດທາງເລືອກ 3:

	ທາງເລືອກ A	ທາງເລືອກ B	ທາງເລືອກເດີມ
ຄຸ້ມຄອງຄ່າກວດດ້ານເຕັກນິກຕ່າງໆ ແລະ ບົ່ງມະຕິພະຍາດ		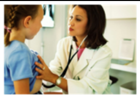	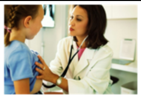
ຄຸ້ມຄອງຄ່າປິ່ນປົວຍ້ອນອຸບັດຕິເຫດຈາກການສັນຈອນ	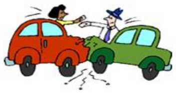		
ຄຸ້ມຄອງຄ່າເດີນທາງໄປໂຮງໝໍຕ່າງເມືອງຂອງຄົນເຈັບ			
ກອງທຶນຄຸ້ມຄອງຄ່າຢາ	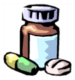	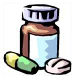	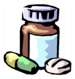
ອັດຕາເງິນສົມທົບ	1 = 12,000<br>2-4 = 20,000<br>5-7 = 25,000<br>≥8 = 28,000 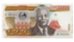	1 = 12,000<br>2-4 = 20,000<br>5-7 = 25,000<br>≥8 = 28,000 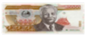 + 4,000	1 = 12,000<br>2-4 = 20,000<br>5-7 = 25,000<br>≥8 = 28,000 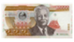
ຄຸ້ມຄອງຄ່ານອນໂຮງໝໍ			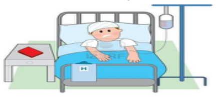
ສ່ວນຫຼຸດສໍາລັບການຈ່າຍເງິນສົມທົບໝົດປີລ່ວງໜ້າ	-5%	-5%	
	☐	☐	☐

ຊຸດທາງເລືອກ 4:

	ທາງເລືອກ A	ທາງເລືອກ B	ທາງເລືອກເດີມ
ຄຸ້ມຄອງຄ່າກວດດ້ານເຕັກນິກຕ່າງໆ ແລະ ບົ່ງມະຕິພະຍາດ	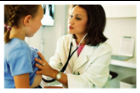	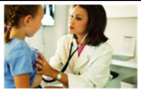	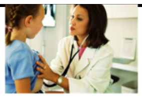
ຄຸ້ມຄອງຄ່າປິ່ນປົວຍ້ອນອຸບັດຕິເຫດຈາກການສັນຈອນ	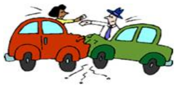		
ຄຸ້ມຄອງຄ່າເດີນທາງໄປໂຮງໝໍຕ່າງເມືອງຂອງຄົນເຈັບ	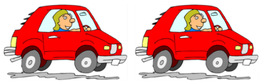	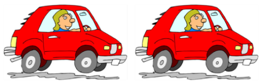	
ກອງທຶນຄຸ້ມຄອງຄ່າຢາ	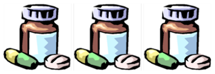	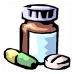	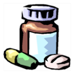
ອັດຕາເງິນສົມທົບ	1 = 12,000<br>2-4 = 20,000<br>5-7 = 25,000<br>≥8 = 28,000 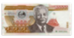 - 2,000	1 = 12,000<br>2-4 = 20,000<br>5-7 = 25,000<br>≥8 = 28,000 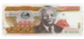 + 4,000	1 = 12,000<br>2-4 = 20,000<br>5-7 = 25,000<br>≥8 = 28,000 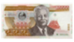
ຄຸ້ມຄອງຄ່ານອນໂຮງໝໍ		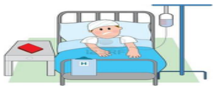	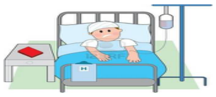
ສ່ວນຫຼຸດສໍາລັບການຈ່າຍເງິນສົມທົບໝົດປີລ່ວງໜ້າ		-5%	
	☐	☐	☐

ຊຸດທາງເລືອກ 5:

	ທາງເລືອກ A	ທາງເລືອກ B	ທາງເລືອກເດີມ
ຄຸ້ມຄອງຄ່າກວດດ້ານເຕັກນິກຕ່າງໆ ແລະ ບົ່ງມະຕິພະຍາດ		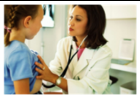	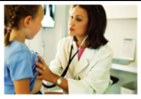
ຄຸ້ມຄອງຄ່າປິ່ນປົວຍ້ອນອຸບັດຕິເຫດຈາກການສັນຈອນ			
ຄຸ້ມຄອງຄ່າເດີນທາງໄປໂຮງໝໍຕ່າງເມືອງຂອງຄົນເຈັບ		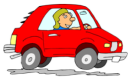	
ກອງທຶນຄຸ້ມຄອງຄ່າຢາ	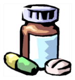	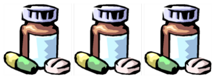	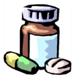
ອັດຕາເງິນສົມທົບ	1 = 12,000<br>2-4 = 20,000<br>5-7 = 25,000<br>≥8 = 28,000<br>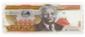<br>+ 4,000	1 = 12,000<br>2-4 = 20,000<br>5-7 = 25,000<br>≥8 = 28,000<br>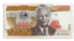<br>- 2,000	1 = 12,000<br>2-4 = 20,000<br>5-7 = 25,000<br>≥8 = 28,000<br>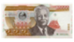
ຄຸ້ມຄອງຄ່ານອນໂຮງໝໍ	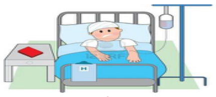	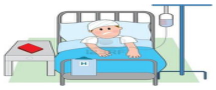	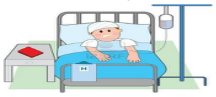
ສ່ວນຫຼຸດສຳລັບການຈ່າຍເງິນສົມທົບໝົດປີລ່ວງໜ້າ	-5%	-5% -5%	
	☐	☐	☐
